# Circulating extracellular vesicles from individuals at high-risk of lung cancer induce pro-tumorigenic conversion of stromal cells through transfer of miR-126 and miR-320

**DOI:** 10.1186/s13046-021-02040-3

**Published:** 2021-07-21

**Authors:** Francesca Pontis, Luca Roz, Mavis Mensah, Miriam Segale, Massimo Moro, Giulia Bertolini, Ilaria Petraroia, Giovanni Centonze, Anna Maria Ferretti, Paola Suatoni, Ugo Pastorino, Orazio Fortunato, Gabriella Sozzi

**Affiliations:** 1 Tumor Genomics Unit, Department of Research, Fondazione IRCCS Istituto Nazionale Dei Tumori, Via Venezian 1, 20133 Milan, Italy; 2 Istituto Di Scienze E Tecnologie Chimiche-CNR, Via G. Fantoli 16/15, 20138 Milan, Italy; 3 Thoracic Surgery Unit, Fondazione IRCCS Istituto Nazionale Dei Tumori, 20133 Milan, Italy

**Keywords:** Extracellular vesicles, microRNA, Lung cancer

## Abstract

**Background:**

Extracellular vesicles (EVs) containing specific subsets of functional biomolecules are released by all cell types and analysis of circulating EVs can provide diagnostic and prognostic information. To date, little is known regarding the role of EVs both as biomarkers and potential key players in human lung cancer.

**Methods:**

Plasma EVs were isolated from 40 cancer-free heavy-smokers classified according to a validated 24-microRNA signature classifier (MSC) at high (MSCpos-EVs) or low (MSCneg-EVs) risk to develop lung cancer. EVs origin and functional properties were investigated using in vitro 3D cultures and in vivo models. The prognostic value of miRNAs inside EVs was assessed in training and in validation cohorts of 54 and 48 lung cancer patients, respectively.

**Results:**

Different membrane composition, biological cargo and pro-tumorigenic activity were observed in MSCpos vs MSCneg-EVs. Mechanistically, in vitro and in vivo results showed that miR-126 and miR-320 from MSCpos-EVs increased pro-angiogenic phenotype of endothelial cells and M2 polarization of macrophage, respectively. MSCpos-EVs prompted 3D proliferation of non-tumorigenic epithelial cells through c-Myc transfer. Moreover, hypoxia was shown to stimulate the secretion of EVs containing c-Myc from fibroblasts, miR-126-EVs from endothelial cells and miR-320-EVs from granulocytes. Lung cancer patients with higher levels of mir-320 into EVs displayed a significantly shorter overall survival in training [HR2.96] and validation sets [HR2.68].

**Conclusion:**

Overall our data provide a new perspective on the pro-tumorigenic role of circulating EVs in high risk smokers and highlight the significance of miR-320-EVs as a new prognostic biomarker in lung cancer patients.

**Supplementary Information:**

The online version contains supplementary material available at 10.1186/s13046-021-02040-3.

## Background

Lung cancer remains the leading cause of cancer-related deaths, despite the substantial advances made over the past 20 years in its prevention, diagnosis and treatment [1]. The 5-year relative survival rate of patients diagnosed with non-small-cell lung cancer (NSCLC), the most common lung cancer subtype (accounting for 80% of lung cancers), is approximately 20% [1] mainly due to delay in diagnosis.

Non invasive biomarkers able to assess cancer risk and improve early diagnosis [2] may impact on patients’ clinical management possibly reducing overall mortality. The largest European randomized lung cancer screening trial, NELSON, also reported a reduction of 25% in lung cancer mortality with low-dose computed tomography (LDCT) screening, compared to no screening at 10 years of follow-up [3]. Recently, the Multicentric Italian Lung Detection (MILD) trial, provided additional evidence that extended intervention beyond 5 years, increased benefit (39% mortality reduction) of screening [4]. However, despite its promising potential and to avoid its high rate of false-positive results (18%), the LDCT screening method requires repeated image acquisition and unnecessary patient exposure to radiation, with a consequent economic burden and patient anxiety [5]. To identify early-stage tumors and small lesions with aggressive potential at early stages or individuals at higher risk of developing aggressive tumors, a better understanding of the processes involved in carcinogenesis is needed. Blood samples are a suitable and easily obtainable source of biomarkers such as circulating cell-free tumor DNA, circulating microRNAs (miRNAs) and, more recently, extracellular vesicles (EVs)[6].

EVs are an evolutionarily conserved group of bilayer membrane vesicles [7, 8]. They are generally classified by size and intracellular origin into small EVs (sEVs, also called exosomes) derived from multivesicular bodies of late endosomes (~ 50–150 nm in diameter) and microvesicles (MVs or ectosomes) that are generated via extracellular membrane budding (~ 100 nm–1 mm in diameter)[9]. EVs contain specific biological functional components, such as proteins, lipids and nucleic acids such as DNA and RNA (messenger RNAs; long noncoding RNAs; miRNAs)[10, 11], that can modulate and influence several biological processes in recipient cells, thus playing a central role in intercellular communication [12–15].

Among the different EV’s cargo components, miRNAs have the largest impact on oncology thanks to their ability to act as both oncogenes and tumor suppressors. MiRNAs are endogenous small non-coding RNAs (21–25 nucleotides) that play a pivotal role in the regulation of gene expression. Deregulation of miRNA expression and the consequent alterations in related biological processes can lead to cancer development, as demonstrated by several studies [16]. Detection of early pro-tumorigenic changes in high-risk individuals is critical for guiding clinical management and improving overall outcomes [2].

In two large prospective studies, we previously assessed the value of circulating miRNAs for early detection of lung cancer in the context of LDCT screening [17, 18]. Notably, we described a lung cancer risk classifier based on the reciprocal ratios of 24 plasma miRNAs (the MiRNA Signature Classifier, MSC) with predictive, diagnostic, and prognostic potential in heavy smokers [17, 18]. Our previous data demonstrated an interesting link between the modulation of these 24 circulating miRNAs and the development of an immunosuppressive and pro-tumorigenic lung microenvironment [19], also substantiating a stromal origin for these ‘risk’ miRNAs. Furthermore, we proved that specific miRNAs composing the MSC signature, miR-486 and miR-660, play a functional role in the modulation of the p53 cancer-associated pathway [20, 21].

Although many studies have elucidated the function of EV-miRNAs released by cancer cells, to date only a few studies have analyzed EV-miRNAs circulating in cancer-free subjects and focused on their interaction with recipient cells to favor a pro-tumorigenic milieu. Our study aimed to characterize plasma EVs isolated from smokers at risk of developing lung cancer and to elucidate their pro-tumorigenic role, focusing on the functional interactions between EV-miRNAs and specific microenvironmental target cells.

## Methods

### Clinical specimens

NPlasma samples were collected from high-risk heavy-smoker volunteers (age 50–75 years), including current or former smokers with a minimum pack/year index of 30 enrolled in a LDCT screening trial performed at our institution (BioMild Trial, ClinicalTrials.gov: NCT02247453)[22]. Clinical characteristics of individuals are summarized in Suppl. Table 1.

EVs were also isolated from the plasma of lung cancer patients affered to Thoracic Unit of our Institute and divided into training (n = 54) and validation (n = 48) set (Suppl. Table 2). These cohorts comprise lung cancer patients dead within 2 years from diagnosis or alive at 5 years. Plasma collection was approved by the Internal Review and the Ethics Boards of the Istituto Nazionale Tumori (INT 11–21) of Milan. All patients provided informed consent.

### In vivo experiment

In vivo experiments were performed with 7–10-week-old female SCID mice (Charles River Laboratories). In brief, 10,000 or 100,000 A549 cells and EV-pretreated HUVECs (1:3) were subcutaneously injected with 1:1 Matrigel:RPMI, and tumor growth was measured weekly using calipers. Animal studies were conducted according to the guidelines of the Ethics Committee for Animal Experimentation of the Fondazione IRCCS Istituto Nazionale dei Tumori (INT_13_2015)[23].

### Statistical analyses

All experiments were performed at least in triplicate, and all values are reported as the mean ± SEM.

The Survival curves were plotted using the Kaplan–Meier method and to compare the two groups of patients we applied the log-rank test. We considered our findings as statistically significant when the p-value was < 0.05. The expression values of miR-320 and miR-126 in survival analysis were clustered as high versus low based on EV-miRNA median expression level. Analyses were performed using GraphPad Prism (GraphPad Software, La Jolla, California USA). Statistical significance was determined using ANOVA with the Tukey’s multiple comparison tests, unpaired or paired t tests. P-values less than 0.05 were considered statistically significant.

## Results

### Isolation and characterization of EVs from heavy-smoking individuals

**Fig. 1:**
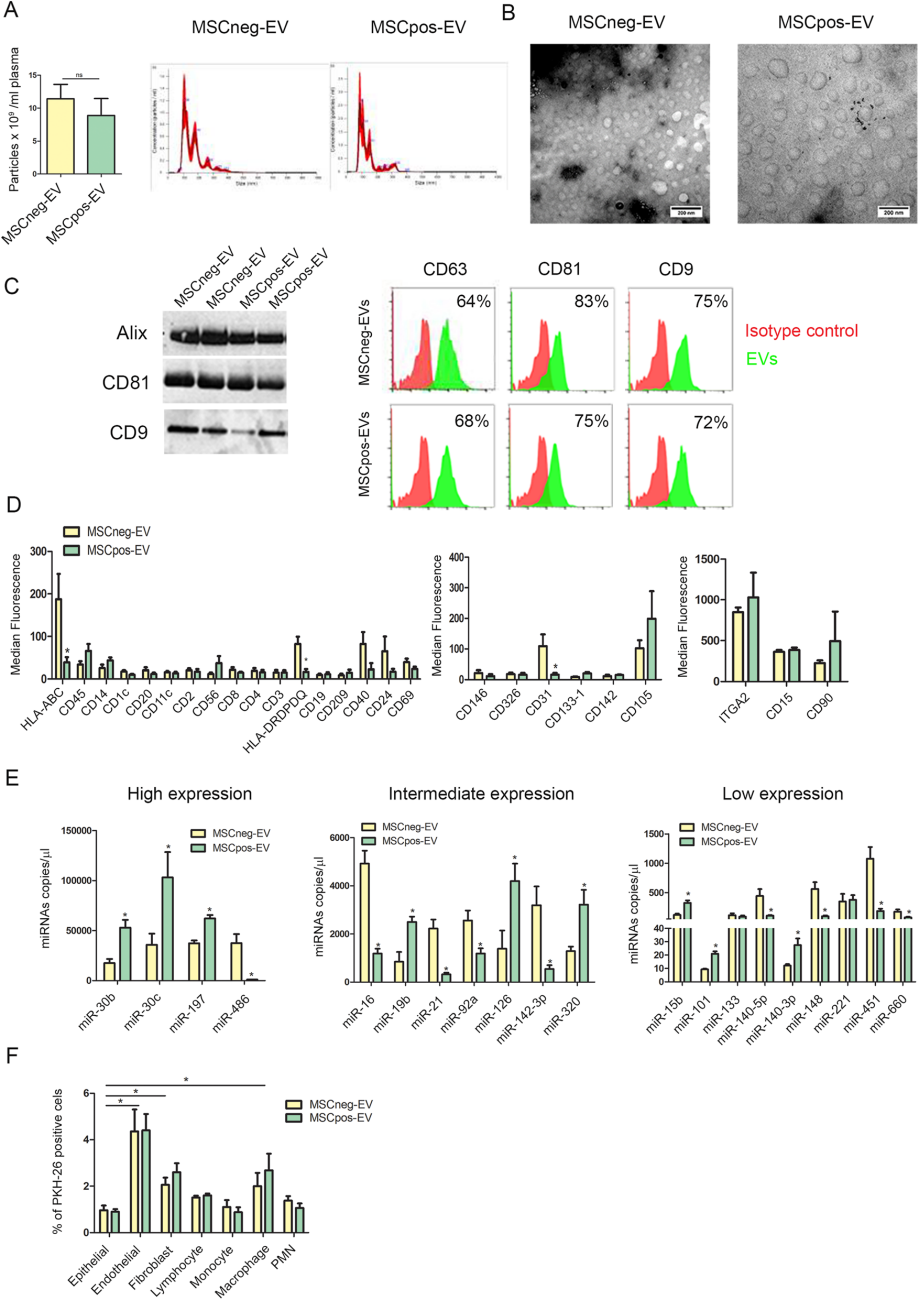
Characterization of plasma heavy-smoking individual-derived EVs. **A)** Concentration and size distribution of plasma-derived EVs from subjects with a low (MSCneg-EVs) or high (MSCpos-EVs) risk of lung cancer using nanoparticle tracking analysis (*n* = 5 per group). **B)** Representative TEM images showing the spherical morphology and size distribution of plasma-derived EVs (red arrows)**. C)** Western blot (left panel) and flow cytometric (right panel) analysis of conventional EV markers on MSCpos- and MSCneg-EVs showing the presence of Alix and the CD63, CD81, and CD9 tetraspanins (*n* = 3 per group). **D)** Profiles of plasma-derived EVs for immune (left), epithelial and endothelial (centre) cell surface markers determined by flow cytometry. Fibroblasts and PMN markers detected on plasma-EVs (right). The values are the median fluorescence intensities (n = 5 per group). **E)** Absolute quantification of 24 miRNAs in EVs from individuals with different levels of lung cancer risk by dPCR (*n* = 5 per group). **F)** Flow cytometric analysis of the percentage of PKH26^+^ cells after treatment with PKH26-labeled MSCpos- and MSCneg-EVs (1 µg) (n = 5 per group). *p < 0.05 versus epithelial cells. The data are expressed as the mean ± S.E.M. values

EVs from heavy-smoking individuals with a low (MSCneg-EVs) or high (MSCpos-EVs) risk of lung cancer [18] were isolated from 1 ml of plasma and characterized in accordance with the Minimal Information for Studies of Extracellular Vesicles (MISEV) guidelines [24]. Nanoparticle tracking analysis did not show significant differences in the number of total circulating particles between the two groups (Fig. 1A). Similar particle size distributions were observed for MSCneg-EVs and MSCpos-EVs (Fig. 1A and Suppl. Table 3), confirming the presence of both sEVs and MVs. TEM analysis of EVs revealed that plasma EVs had spherical shapes and a relatively wide size distribution (Fig. 1B and Suppl. Figure 1A). The Western blot results showed that both MSCpos- and MSCneg-EVs were positive for conventional EV markers such as CD9, CD81 and Alix (Fig. 1C, left). Flow cytometric analysis further confirmed the expression of the tetraspanins CD9, CD63, and CD81 on the surface of the EVs (Fig. 1C, right and Suppl. Figure 1B).

We performed multiplex phenotypic analysis using the MACSPlex platform to reveal the cell-type specific origin of EVs isolated from heavy-smoking individuals. This analysis showed that circulating EVs in heavy-smoking individuals were derived mainly from immune cells, as indicated by the expression of hematopoietic markers (CD3-,CD11c-, HLA-DR-, CD45-positive) (Fig. 1D, left), although EVs derived from epithelial cells (CD326-positive) and endothelial cells (CD31- and CD105-positive) were also observed (Fig. 1D, center). Interestingly, plasma EVs were also secreted by fibroblasts (integrin-α2- and CD90-positive) and PMNs (CD15-positive) (Fig. 1D, right).

The results of dPCR with absolute quantification of 24 miRNAs composing MSC signature in EVs revealed that 9 miRNAs were upregulated (miR-15b, miR-19b, miR-30b, miR-30c, miR-101, miR-126, miR-140-3p, miR-197 and miR-320), 13 were downregulated (miR-16, miR-17, miR-21, miR-28-3p, miR-92a, miR-106, miR-140-5p, miR-142-3p, miR-145, miR-148, miR-451, miR-486, and miR-660), and 2 (miR-133 and miR-221) were not modulated in MSCpos-EVs compared to MSCneg-EVs (n = 5 per group) (Fig. 1E and Suppl. Figure 1C). In this set, we observed an 83.3% correlation between the modulation of 24-miRNA content in EVs and plasma in high-risk subjects. The up-regulation of two host-related miRNAs, miR-126 and miR-320 in MSCpos-EVs was further confirmed in a validation set of 30 healthy heavy-smoking individuals (Suppl. Figure 1D).

To investigate the potential pro-tumorigenic activity of plasma EVs, we initially evaluated the in vitro interaction between EVs from both MSCpos and MSCneg individuals and different cells composing the lung microenvironment, such as endothelial cells, fibroblasts, immune cells and human bronchial epithelial cells (HBECs), recently included as a potential component of the TME [25]. To this end, PKH26-labeled EVs (corresponding to 1 µg of total protein) were incubated with different cell types. Flow cytometric analysis showed a preferential uptake of EVs by certain types of lung micro environmental cells such as endothelial cells, fibroblasts and macrophages compared to other cell types such as epithelial, monocytes, polymorphonuclear cells (PMN). No differential uptake was observed between MSCpos-EV and MSCneg-EVs (Fig. 1F).

Having demonstrated that plasma EVs are preferentially up taken by endothelial cells, fibroblasts and macrophages, we next functionally assess their potential pro-tumorigenic effects on these cells compartments, in particular focusing on the difference in miRNA cargo that discriminate MSCpos or MSCneg EVs.

### MiR-126 in MSCpos-EVs modulates the proangiogenic ability of endothelial cells

**Fig. 2:**
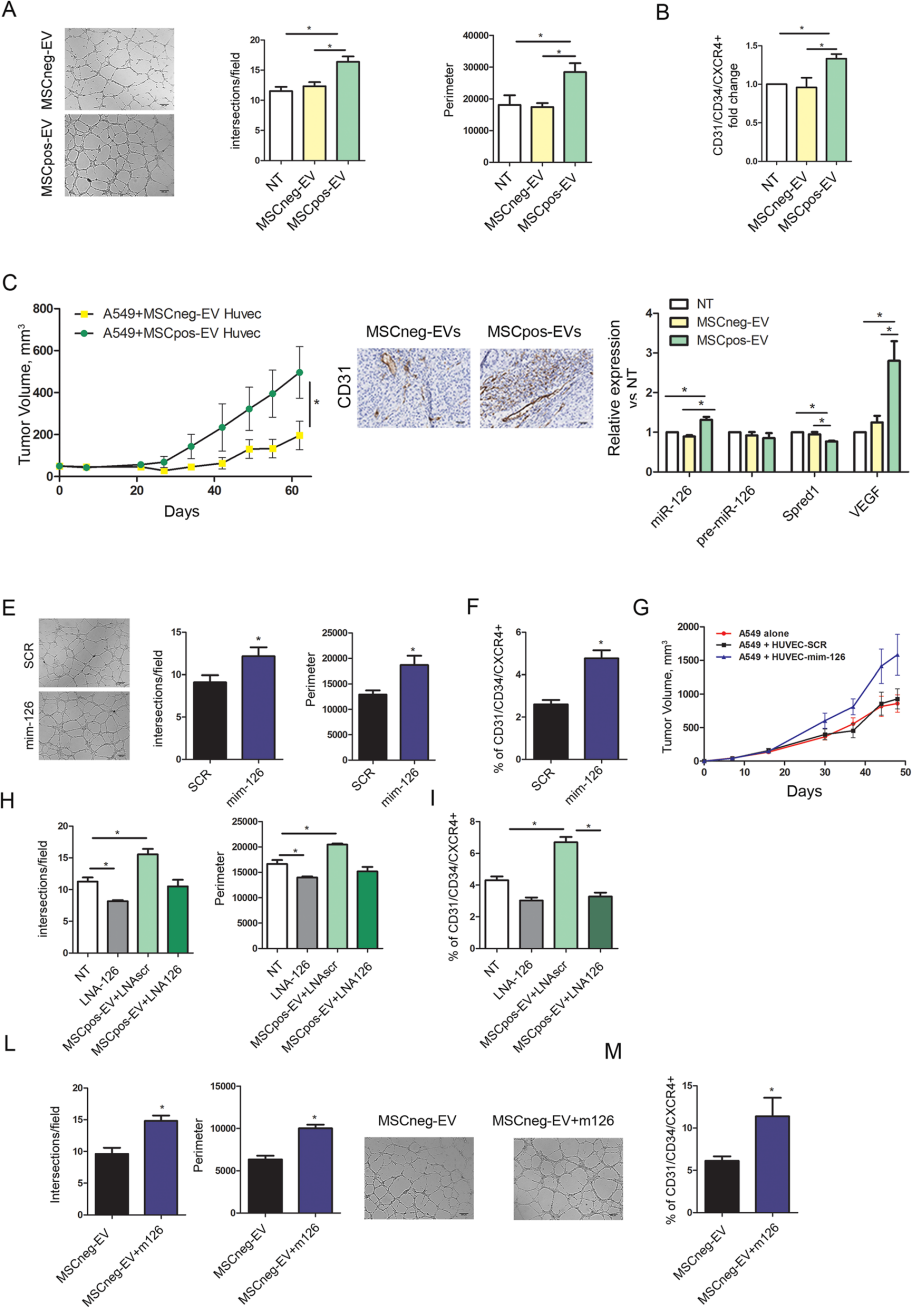
MSCpos-EVs modulate the phenotype and angiogenic ability of endothelial cells. **A)** Quantification of the number of intersections and network perimeters formed by HUVECs on the Matrigel layer after MSCpos- and MSCneg-EV treatment. Untreated cells were used as a control. (n = 5 per group). **B)** Flow cytometric analysis of the activated HUVEC phenotype (CD31^+^/CD34^+^/CXCR4^+^) after MSCpos- and MSCneg-EV treatment. Untreated cells (NT) were used as a control (*n* = 5). **C)** Tumor growth curves and CD31 IHC staining of 10^4^ lung cancer (A549) cells coinjected with HUVECs pretreated with MSCpos- and MSCneg-EVs in immunodeficient mice (n = 7 for each group). **D)** Analysis of the relative expression levels of miR-126, pre-miR-126, SPRED1 and VEGF in HUVECs treated with MSCpos- and MSCneg-EVs. Untreated cells (NT) were used as a control (n = 5). **E)** Tube formation assay of miR-126-overexpressing HUVECs compared to control SCR-expressing HUVECs on Matrigel (*n* = 5). **F**) The graphs show the percentages of the CD31^+^/CD34^+^/CXCR4^+^ population among miR-126-overexpressing cells (*n* = 5) and (**G**) the in vivo growth curves of 10^5^ A549 cells coinjected with the same HUVECs (*n* = 5 mice per group). **H)** Cells pretransfected with LNA 126 were incubated with MSCpos-EVs, and capillary-like structure formation was analyzed (*n* = 4). **I)** The panel shows a reduction in the CD31^+^/CD34^+^/CXCR4^+^ HUVEC-LNA126 population after treatment with MSCpos-EVs compared to the corresponding control treatment (n = 4). **L)** Vessel network formation and **M)** activation of endothelial cells after treatment with MSCneg-EVs and MSCneg-EVs enriched with mimic-126 (*n* = 5). *p < 0.05 versus controls. The data are expressed as the mean ± S.E.M. values

The functional role of EVs from MSCpos individuals was also explored on specific components of the TME, and changes in the endothelial cell phenotype after EVs treatment were analyzed. First, treatment with MSCpos-EVs increased the ability of HUVECs to form tubular structures compared to MSCneg-EVs treated and untreated (NT) HUVECs (number of intersections: NT, 11.5 ± 0.7; MSCneg-EVs, 12.3 ± 0.7; MSCpos-EVs: 16.4 ± 0.9; p = 0.002) (Fig. 2A). Upregulation of CD34 and CXCR4, which play a role in the activation of endothelial cells towards to a pro-angiogenic phenotype [26, 27], was observed in HUVECs treated with MSCpos-EVs compared to HUVECs treated with MSCneg-EVs (*n* = 5, Fig. 2B and Suppl. Figure 2A). Next, to investigate the impact of endothelial cell-inducing signals on tumor growth, coinjection of EV-treated HUVECs with 10^4^ A549 lung cancer cells was performed in vivo. Interestingly, compared to MSCneg-EV-treated HUVECs, MSCpos-EV-treated HUVECs efficiently supported tumor growth (A549 tumor growth: MSCneg-EVs, 137.5 ± 54 mm^3^; MSCpos-EVs, 364.9 ± 104.4 mm^3^, n = 7 for each group, *p* < 0.05) (Fig. 2C). Furthermore, IHC analysis of tumors revealed an increase of murine CD31positive vessels in MSCpos-EVs group compared to MSCneg-EVs suggesting a greater recruitment of murine endothelial cells by MSCpos-EV-treated HUVEC (Fig. 2C and Suppl. Figure 2B). Since one of the upregulated miRNAs in EVs from MSCpos individuals, miR-126, is well known to be a pro-angiogenic miRNAs on these recipient cells [28–30], we investigated the potential role of miR-126 in the modulation of HUVEC phenotype. First, we evaluated the transfer of this miRNA into endothelial cells via EVs and observed an increase in the miR-126 level (1.2-fold increase, *p* = 0.0079) in HUVECs treated with MSCpos-EVs compared to controls (Fig. 2D), without any difference in the transcription of pre-miR-126 (Fig. 2D). The transfer of miR-126 from MSCpos-EVs was demonstrated by the downmodulation of the Spred1 transcript, a known direct target of miR-126 [28, 29], and up-regulation of VEGF in recipient cells (Fig. 2D).

To demonstrate that miR-126 is implicated in autocrine proangiogenic modulation of the HUVEC phenotype observed after treatment with MSCpos-EVs, we directly modulated miR-126 expression using miRNA mimics (Suppl. Figure 2C). A nontargeting miRNA sequence (SCR) was used as control. Compared to SCR, miR-126 overexpression increased the ability of HUVECs to form capillary-like structures (Fig. 2E) and the expression of CXCR4 and CD34 (n = 5, Fig. 2F and Suppl. Figure 2D). To confirm our in vitro observations, subcutaneous coinjection of immunodeficient mice with miR-126-overexpressing HUVECs and 10^5^ A549 cells showed that these cells significantly enhanced in vivo tumor growth compared to that resulting from treatment with SCR-expressing HUVECs (A549 tumor growth: SCR, 1010 ± 124 mm^3^; mim-126, 1774 ± 291.4 mm^3^, p < 0.05), with the same effect as that seen in MSCpos-EV-treated HUVECs (Fig. 2G). HUVEC-mim-126 were able to increase the recruitment of murine endothelial cells to substain tumor growth as shown in Suppl. Figure 2E.

Moreover, the observed proangiogenic role of miR-126 was thoroughly confirmed by transfecting HUVECs with an anti-miR-126 LNA that inhibits miR-126 shuttling before the addition of MSCpos-EVs (Suppl. Figure 3A). MSCpos-EVs stimulated the formation of tube structures (Fig. 2H) and the activation of endothelial cells (n = 4, Fig. 2I and Suppl. Figure 3B), which were abolished in cells treated with miR-126 LNA. Additionally, the importance of this miRNA in angiogenesis was further confirmed by a significant reduction in vessel tubes after miR-126 downmodulation (Fig. 2H).

Finally, the role of this miRNA in angiogenesis was further corroborated by transfecting MSCneg-EVs with miR-126 mimics (Suppl. Figure 3C). Compared to MSCneg-EVs, MSCneg-mim-126-EVs induced vessel network formation (Fig. 2L) and upregulated the surface expression of CD34 and CXCR4 in HUVECs (n = 5, Fig. 2M and Suppl. Figure 3D).

Overall, these data demonstrate that miR-126 in EVs isolated from MSCpos individuals can stimulate a proangiogenic process with potential functional consequences in the lung microenvironment.

### MSCpos-EVs induce M2 polarization of macrophages through PMN released miR-320

**Fig. 3:**
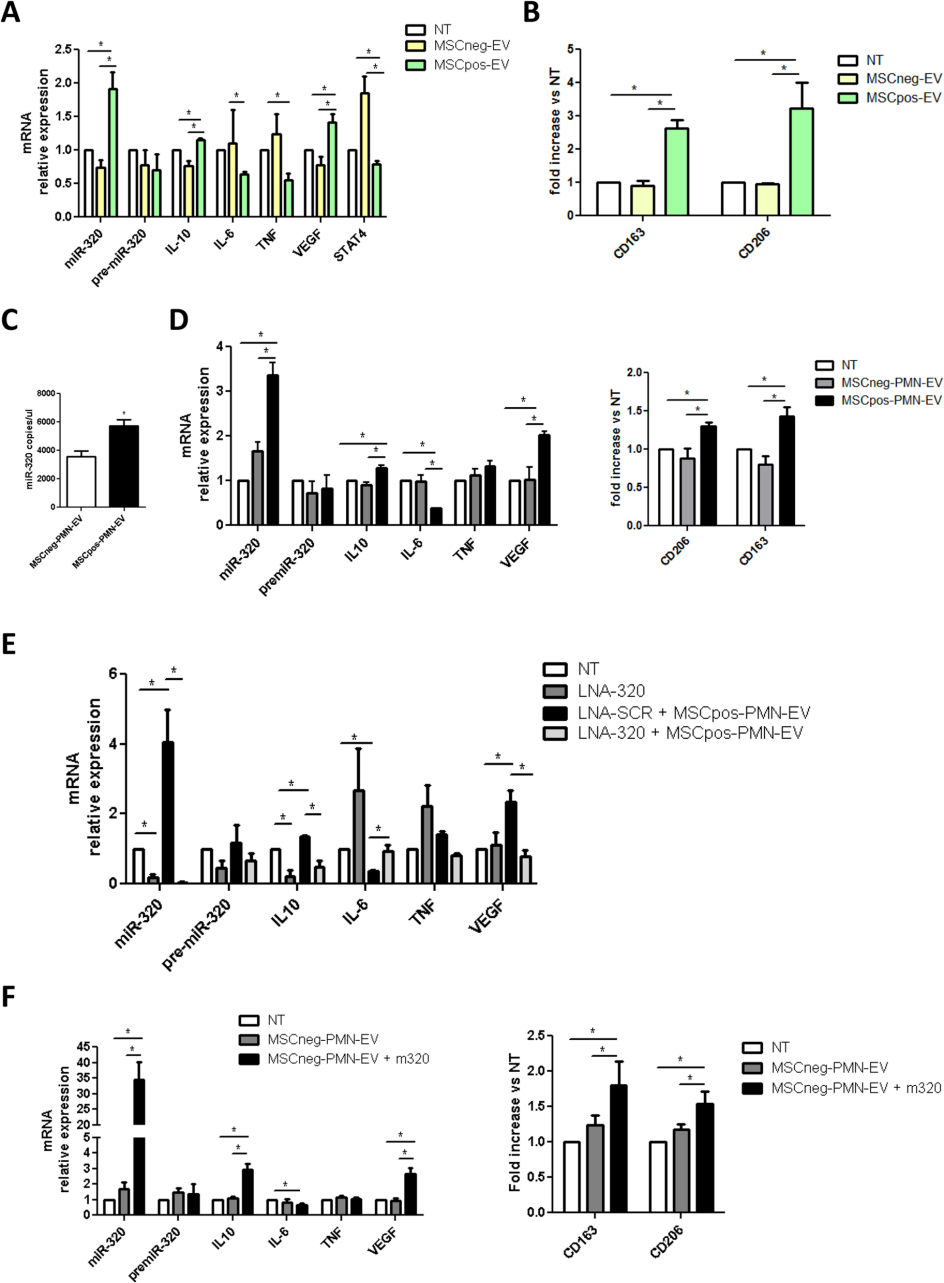
MSCpos-EVs induce M2 polarization of macrophages. **A)** Intracellular expression levels of miR-320, pre-miR-320 and M2 phenotype markers in macrophages treated with MSCpos- and MSCneg-EVs, as measured by qPCR. Untreated cells were used as a control (*n* = 5). **B)** Flow cytometric analysis of CD163 and CD206 expression in macrophages treated with MSCpos- and MSCneg-EVs (*n* = 5). **C)** Absolute quantification (copies/µl) of the miR-320 content in PMN-EVs isolated from MSCpos and MSCneg individuals (*n* = 9). **D)** Relative expression levels of miR-320, pre-miR-320, and M2 mRNA (**center**), as measured by qPCR, and CD163 and CD206 levels, as measured by flow cytometric analysis (**right**) of macrophages treated with MSCpos- and MSCneg-PMN-EVs (*n* = 4). **E)** Analysis of relative miRNA and mRNA expression levels in macrophages transfected with LNA-320 and treated with MSCpos-EVs (*n* = 4). **F)** Analysis of miR-320, mRNA and CD163/CD206 expression levels in macrophages treated with MSCneg-PMN-EVs enriched with the miRNA-320 mimic (*n* = 5). Untreated (NT) macrophages were used as a control. *p < 0.05 versus controls. The data are expressed as the mean ± S.E.M. values

To clarify the potential role of EVs and their miRNA cargo in modulating the development of a protumorigenic microenvironment, we focused on investigating their effect on macrophages. Macrophages obtained from PBMC of heavy-smoking individuals were treated for 72 h with EVs from both MSCpos and MSCneg individuals, and the expression of the cytokines IL-10, IL-6, TNF-a and VEGF was analyzed by RT-PCR to evaluate skewing towards a Th1 or a Th2 response. In MSCpos-EV-treated macrophages, intracellular IL-10 (fold increase 1.16 ± 0.02, p = 0.0025) and VEGF upregulation (fold increase: 1.42 ± 0.11, *p* = 0.0375) coupled with IL-6 (fold decrease: 0.67 ± 0.04, *p* = 0.0017) and TNF-α (fold decrease: 0.55 ± 0.09, *p* = 0.018) downmodulation were observed (Fig. 3A). The modulation of cytokines after MSCpos-EVs treatment was confirmed by ELISA on CM and lysates of macrophages (Suppl. Figure 4A). Flow cytometric analysis showed an increase in CD163- and CD206-positive cells after MSCpos-EVs addition (Fig. 3B and Suppl. Figure 4B). Upregulation of miR-320, which we and others previously reported to be released by PMNs and to induce M2 polarization[19, 31], was observed in MSCpos-EVs compared to MSCneg-EVs (Fig. 1E). Transfer of miR-320 in macrophages was confirmed by the increase in the level of this miRNA and downmodulation of its target STAT4, which occurred without modulation of pre-miR-320 levels (Fig. 3A). STAT4 protein de-regulation in MSCpos-EV treated macrophages was also confirmed by Western Blot analysis (Suppl. Figure 4C).

To further explore the role of miR-320 in EVs, EVs were isolated from PMNs of MSCpos (MSCpos-PMN-EVs) and MSCneg (MSCneg-PMN-EVs) individuals, and the results demonstrated that the level of this miRNA was significantly higher in MSCpos-PMN-EVs than in MSCneg-PMN-EVs (miR-320 copies/µl: 5724.9 and 3514.2 in MSCpos-PMN-EVs and MSCneg-PMN-EVs, respectively, *p* = 0.0033) (Fig. 3C). To assess the role of miR-320 in PMN-derived EVs in modulating macrophage polarization, we treated macrophages with MSCpos- and neg-PMN-EVs. RT-PCR showed upregulation of IL-10 and VEGF, with IL-6 downmodulation, in macrophages after treatment with MSCpos-PMN-EVs compared to after treatment with MSCneg-PMN-EVs (Fig. 3D). Interestingly, direct transfer of miR-320 from MSCpos-PMN-EVs to macrophages was then observed, without modulation of the pre-miR-320 transcript levels (Fig. 3D). MSCpos-PMN-EVs were able to induce the up-regulation of CD163 and CD206 on the surface of macrophages evaluated by flow cytometry (Fig. 3D and Suppl. Figure 5A). The immunosuppressive role of miR-320 released by PMNs was assessed by using LNA-miR-320 to inhibit miR-320 function in recipient cells. As shown in Fig. 3E, the upregulation of IL-10 and VEGF and downmodulation of IL-6 in MSC-PMN-EV-treated macrophages was abolished in macrophages with inhibited miR-320.

To further elucidate the role of miR-320-EVs in M2 polarization of macrophages, a miR-320 mimic was transfected into MSC-neg-PMN-EVs (Suppl. Figure 5B), and changes in the treated cells were evaluated. EVs with high miR-320 levels induced upregulation of IL-10 and VEGF coupled with downmodulation of IL-6, suggesting M2 polarization (Fig. 3F). This immunosuppressive effect was also confirmed by the increase in CD163- and CD206-positive macrophages (Fig. 3F and Suppl. Figure 5C).

### c-Myc-enriched-MSCpos-EVs induce pro-tumorigenic changes on bronchial epithelial cell

**Fig. 4:**
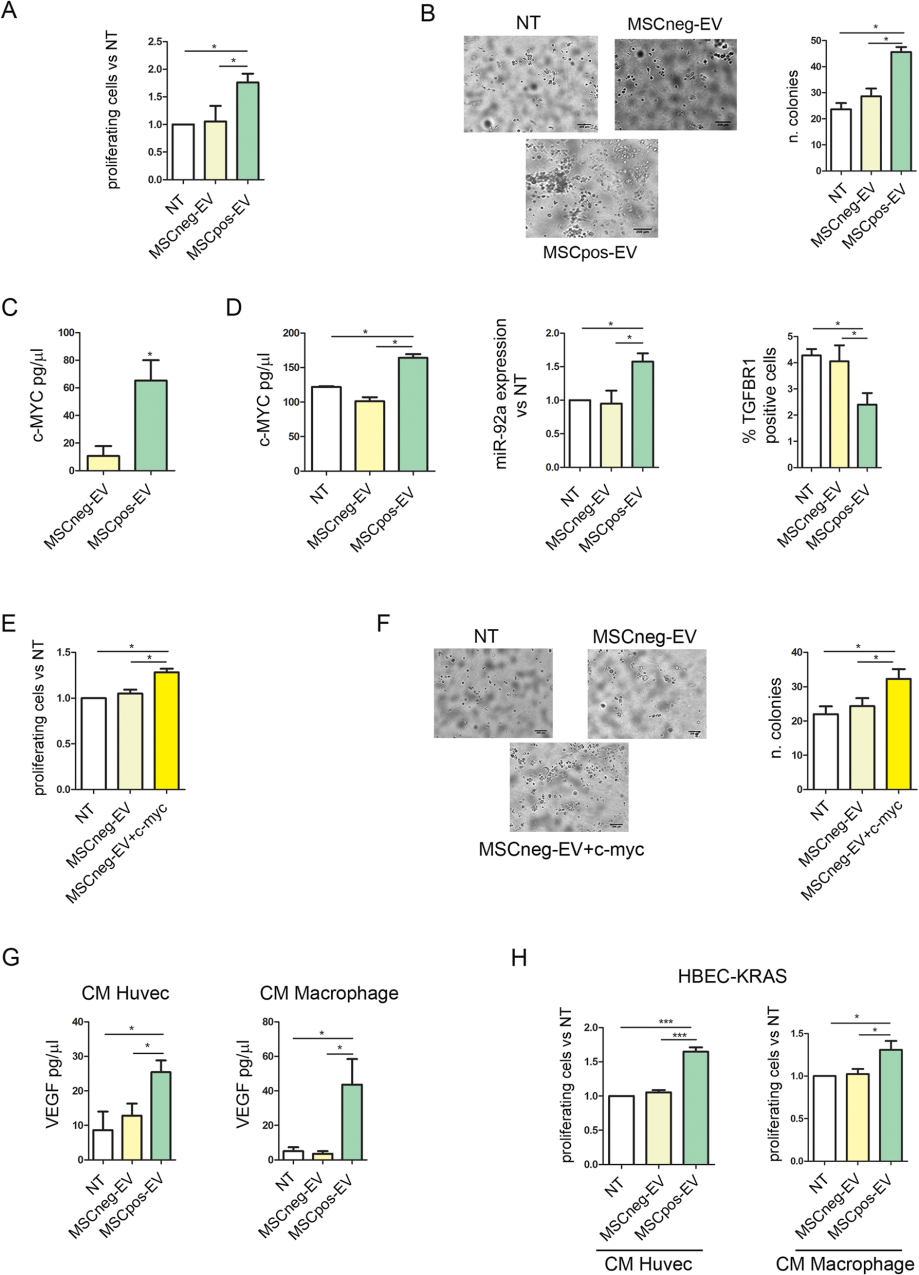
MSCpos-EV treatment increases epithelial bronchial cell proliferation. **A)** Bar plots showing the viability rate of HBEC-KRAS^V12high^ cells after MSCpos- and MSCneg-EV treatment, as assessed by a RealTime-Glo assay (*n* = 5). **B)** Analysis of the ability of HBEC-KRAS^V12high^ cells to grow in 3D culture using VitroGel. The graph shows the number of colonies formed after 2 weeks by cells treated with MSCpos- and MSCneg-EVs (*n* = 5). **C)** Quantification of the c-Myc content in MSCpos- and MSC-neg-EVs (*n* = 5). **D)** Histograms showing the intracellular c-Myc content (**left**), miR-92a levels (**center**) and TGFBRI (**right**) in HBEC-KRAS^V12high^ cells after MSCpos- and MSCneg-EV treatment (*n* = 4). **E)** Proliferation analysis of HBEC-KRAS^V12high^ cells treated with MSCneg-EVs overexpressing c-Myc (*n* = 4). **F**) Representative images and bar graphs showing the ability of HBEC-KRAS^V12high^ cells treated with MSCneg-EVs + c-Myc to grow in 3D culture compared to that of cells treated with MSCneg-EVs (number of colonies)(*n* = 3). **G)** Analysis of VEGF-A levels (pg/ml) in CM collected from HUVECs and macrophages after 48 h of treatment with MSCpos- and MSCneg-EVs. **H)** Graphs showing the proliferation of HBEC-KRAS^V12high^ cells treated with CM from HUVECs (left panel) and macrophages (right panel) (*n* = 5). Untreated (NT) cells were used as a control. *** *p* < 0.0001, *p < 0.05 versus controls. Data are expressed as the mean ± S.E.M. values

To investigate the potential of MSCpos-EVs to induce a fully tumorigenic phenotype of transformed bronchial epithelial cells, HBEC-KRAS^V12high^ cells, we first verified the EVs uptake by these cells. These cells show a higher uptake of PKH26-labelled-EVs compared to HBEC-1 counterpart without differences between MSCpos- and MSCneg-EVs (Suppl. Figure 6A). Then, HBEC-KRAS^V12high^ cells were treated with 15 µg of EVs isolated from both MSCpos and MSCneg individuals. Untreated cells (NT) were used as an additional control. Compared with MSCneg-EV-treated cells and NT control cells, MSCpos-EV-treated HBEC-KRAS^V12high^ cells showed a significant increase in their proliferation ability at 72 h (Fig. 4A). Furthermore, we evaluated the ability of EVs to support 3D cell growth. Thus, MSCpos-EV-treated HBEC-KRAS^V12high^ cells were seeded on VitroGel as previously described [32], and enhanced 3D proliferation was observed after 14 days compared to that of MSCneg-EV-treated and NT control cells (Fig. 4B, number of colonies: NT, 23.6 ± 2.3; MSCneg-EVs, 28.6 ± 3; MSCpos-EVs, 45.6 ± 1.9, *p* = 0.0014).

Furthermore, higher levels of the oncoprotein c-Myc, which we previously described as being involved in the malignant transformation of HBEC-KRAS^V12high^ cells [32], were observed in MSCpos-EVs than in MSCneg-EVs (Fig. 4C, c-Myc pg/µl: MSCneg-EVs, 10.6 ± 7.1; MSCpos-EVs, 65.3 ± 14.7, *p* = 0.01). Moreover, this transcription factor was shuttled from MSCpos-EVs to recipient cells (Fig. 4D, HBEC-KRAS^V12high^ c-Myc pg/ml: NT, 121.9 ± 0.9; MSCneg-EVs, 101.2 ± 5.8; MSCpos-EVs, 164.2 ± 5.3, *p* < 0.0001). c-Myc in MSCpos-EVs induced downmodulation of TGFBRI via overexpression of miR-92a in recipient cells (Fig. 4D). The role of c-Myc in the reduction of TGFBRI was corroborated by the absence of the down-modulation of this receptor in LNA-92-treated HBEC-KRAS^V12high^ after MSCpos-EVs administration (Suppl. Figure 6B).

A critical aim was to prove that the phenotypic changes in HBEC-KRAS^V12high^ cells observed after MSCpos-EVs treatment were caused by c-Myc transfer. To this end, the c-Myc plasmid was transfected into MSCneg-EVs, and the EVs were analyzed for their ability to promote the proliferation of HBEC-KRAS^V12high^ cells in both 2D and 3D culture. As shown in Fig. 4E, transfection of c-Myc into MSCneg-EVs increased the proliferation of HBEC-KRAS^V12high^ cells, as evaluated using RealTime-Glo assay. The importance of c-Myc in the EVs was further confirmed by the increase in the number of colonies of HBEC-KRAS^V12high^ cells after treatment with MSCneg-EVs -overexpressing c-Myc compared to that in cells treated with MSCneg-EVs or NT control cells (Fig. 4F, number of colonies: NT, 22 ± 2.3; MSCneg-EVs, 24.3 ± 2.3; MSCneg-EV + c-Myc, 32.3 ± 2.8, *p* = 0.03).

To elucidate the tumor-promoting activity of endothelial cells and macrophages after MSCpos-EVs treatment, CM from HUVECs or macrophages treated for 72 h with MSCpos- and MSCneg-EVs was analyzed. The results revealed that MSCpos-EVs stimulated the secretion of VEGF-A, a factor known to increase epithelial cell proliferation, by both cell types (Fig. 4G). Furthermore, to exclude that the increase of VEGF in MSCpos-EVs treated cells was due to a higher presence of VEGF inside EVs, we measured the levels of this cytokines in EVs lysates. As shown in Suppl. Figure 7 VEGF was present in EVs at very low levels (VEGF pg for 15ug EVs: 5.5 and 4.1 pg of in MSCneg and MSCpos) in both groups. Moreover, the addition of CM from HUVECs treated with MSCpos-EVs to HBEC-KRAS^V12high^ cells increased the number of proliferating cells compared to that resulting from the addition of CM from HUVECs treated with MSCneg-EVs (Fig. 4H), proliferation (proliferating cells vs NT fold increase: CM-HUVEC-MSCneg-EVs, 1.05 ± 0.03; CM-HUVEC-MSCpos-EVs, 1.65 ± 0.06, *p* < 0.0001). Interestingly, CM from macrophages treated with MSCpos-EVs stimulated the growth of HBEC-KRAS^V12high^ cells more effectively than CM from macrophages treated with MSCneg-EVs (Fig. 4H).

### Circulating plasma EVs are secreted by different cellular components of the lung microenvironment

**Fig. 5:**
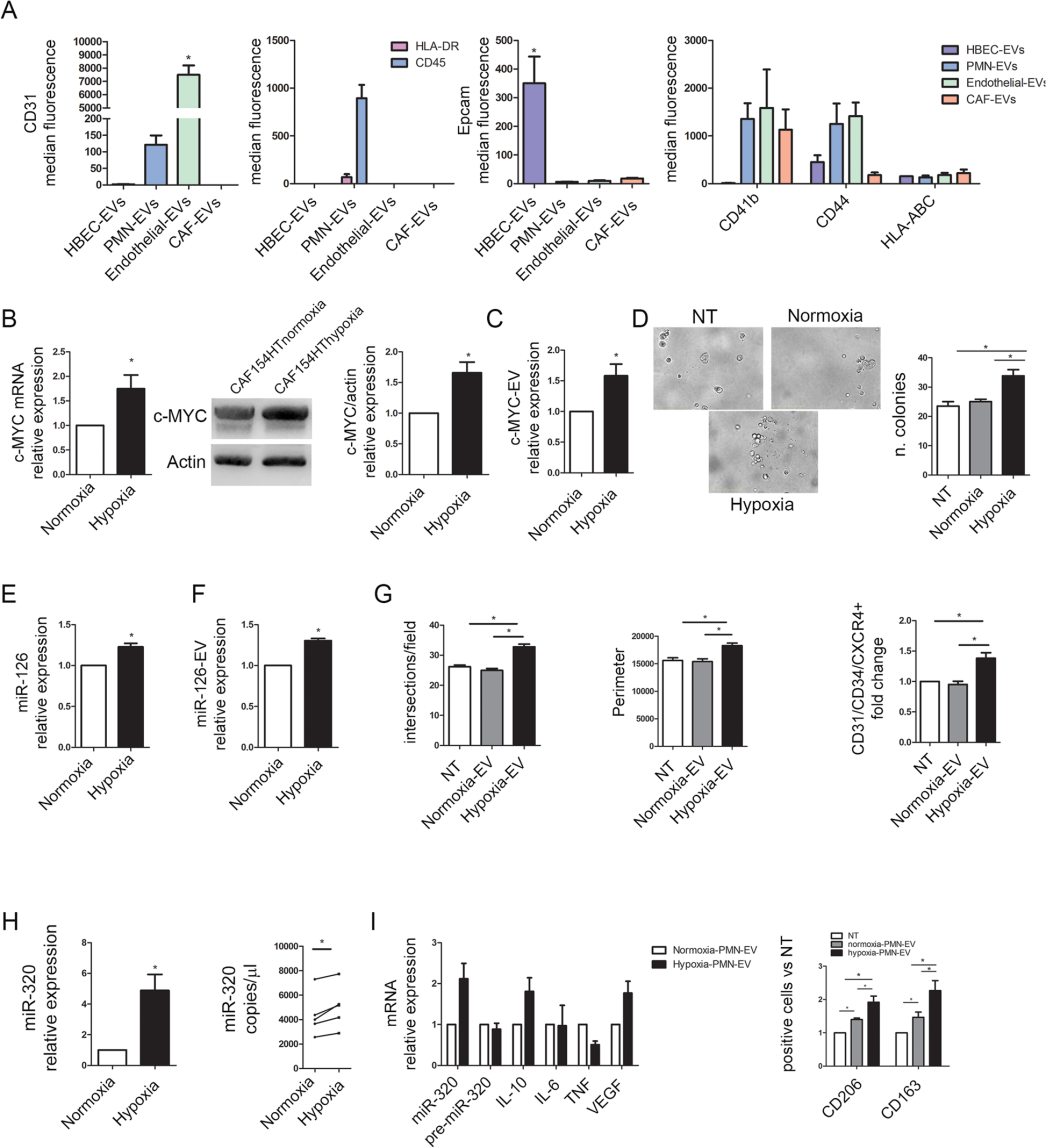
Hypoxia alters the cargo of EVs. **A)** Multiplex phenotypic analysis by flow cytometry (expressed as the median fluorescence intensity) showed the differential expression pattern of membrane proteins on EVs collected from CAFs, PMNs, HUVECs and HBEC-KRAS^V12high^ cells (*n* = 5). **B)** Relative c-Myc mRNA expression levels (left) and protein content (WB, right) in fibroblasts under normoxic or hypoxic (2% O_2_) conditions (*n* = 5). **C)** Quantification of c-Myc protein content in EVs released by normoxic and hypoxic fibroblasts (*n* = 5). **D)** Representative images and analysis results of HBEC-KRAS^V12high^ colony formation in VitroGel after treatment with normoxic and hypoxic fibroblast-derived EVs. **E)** Relative mir-126 expression levels in HUVECs exposed to normoxia or hypoxia (2% O_2_) (*n* = 3). **F)** Quantification of miR-126 in EVs released by normoxic and hypoxic HUVECs (*n* = 3). **G)** Analysis of tube formation and endothelial cell activation after treatment with EVs derived from HUVECs under normoxic or hypoxic conditions (*n* = 5). **H)** Relative expression levels of intracellular miR-320 in PMNs cultured for 24 h under hypoxic (2% O_2_) or normoxic conditions (left) and in EVs (right) (*n* = 5). **I)** Analysis of miRNA-320 and M2 polarization gene mRNA expression levels (left) and CD163/CD206 expression levels by flow cytometry (right) in macrophages treated with EVs released from PMNs cultured under normoxic and hypoxic conditions (*n* = 5). Untreated cells (NT) were used as a control. *p < 0.05 versus controls. The data are expressed as the mean ± S.E.M. values

To investigate the potential origin of circulating plasma EVs we performed a multiplex phenotypic analysis of the EVs surface profile of fibroblasts, endothelial cells, PMNs and epithelial cells. Firstly, we observed that endothelial cell-derived EVs were positive for CD31 (Fig. 5A, *p* < 0.05 compared to other EVs) whereas PMN-derived EVs exclusively displayed hematopoietic-specific markers, as CD45 and human leukocyte antigen (HLA)-DR (Fig. 5A, p < 0.05 compared to other EVs). Epcam was present only on the surface of EVs from epithelial cells (HBEC-KRAS^V12high^) whereas all the analyzed EVs were positive for HLA-ABC and integrins (Fig. 5A).

Comparison of these observations with data obtained from plasma of high-risk individuals (Fig. 1D) indicates that multiple cell types likely contribute to circulating EV’s patterns. In particular, the observed modulation of CD31 or HLA-ABC and DR on MSCpos-EVs could be potentially explained by a different EVs release by endothelial and immune cells respectively. Furthermore, the presence of Epcam (CD326) only on the surface of EVs from HBEC-KRAS^V12high^ suggests that the plasma circulating EVs expressing Epcam may have an epithelial origin.

### *Hypoxia induces *in vitro* modulation of EVs cargo*

We then wanted to investigate the potential mechanisms subtending modulation of EV cargo in high-risk individuals. Since smoking is the major risk factor for the development of lung cancer, and chronic exposure to cigarettes is associated with airflow obstruction with an increase in alveolar hypoxia [33], we carefully investigated whether some of the observed alterations in EVs released in high-risk individuals could be related to specific changes in EVs produced by different cell types under hypoxic stimulation. Based on our findings and previously published studies we analyzed the presence of c-myc in fibroblast [34], miR-126 in endothelial cells[28–30] and miR-320 in PMN cells [19, 31] after hypoxia stimulation.

To this aim, fibroblasts were cultured for 24 h in 2% O_2,_ and we observed an increase in the c-Myc mRNA (fold increase in hypoxic fibroblasts: 1.75 ± 0.28, p = 0.044) and protein (hypoxic c-Myc protein fold increase: 1.66 ± 0.17, *p* = 0.019) levels compared to those in normoxic cells (Fig. 5B). Upregulation of the c-Myc oncoprotein was also observed in EVs released by hypoxic fibroblasts (hypoxic c-Myc-EVs fold increase (pg/ml): 1.58 ± 0.2, *p* = 0.0376) (Fig. 5C), and these EVs supported the 3D growth of HBEC-KRAS^V12high^ cells compared to that observed in cells treated with normoxic EVs or NT cells (Fig. 5D, number of colonies: NT, 23.5 ± 1.5; normoxic EVs, 25 ± 0.9; hypoxic EVs, 33.8 ± 2.1, *p* = 0.003).

Additionally, our results demonstrated that hypoxia stimulated the transcription of miR-126 in endothelial cells (hypoxic miR-126 fold increase: 1.23 ± 0.04, *p* = 0.035) (Fig. 5E) and subsequently upregulated miR-126 in hypoxic EVs (hypoxic miR-126-EVs fold increase: hypoxic EVs, 1.30 ± 0.03; *p* = 0.008) (Fig. 5F). Compared to miR-126 in normoxic EVs, miR-126 in hypoxic EVs stimulated capillary-like structure formation and endothelial cell activation (Fig. 5G). The role of miR-126 in hypoxic-EVs was further confirmed by transfecting HUVECs with an anti-miR-126 LNA that inhibits miR-126 shuttling before the addition of EVs. Hypoxia-EVs stimulated the formation of tube structures which was abolished in cells treated with miR-126 LNA (Suppl. Figure 8A).

Finally, miR-320 was upregulated in PMNs cultured for 24 h in hypoxia (hypoxic miR-320 fold increase: 4.88 ± 1.1, *p* = 0.0167), and these cells secreted EVs with higher levels of this miRNA than normoxic PMNs (Fig. 5H). High levels of miR-320 expression induced an M2-like phenotype in macrophages, as indicated by the increased levels of IL-10, VEGF, CD163 and CD206 (Fig. 5I and Suppl. Figure 8B).

### MiR-320-EVs is a prognostic biomarker in lung cancer patients

We reported previously that circulating levels of miR-320 and miR-126 in plasma of lung cancer patients remain de-regulated after tumor removal [19] suggesting that these two miRNAs could be considered “host-related miRNAs” and reflect the persistence of an elevated risk profile.

**Fig. 6:**
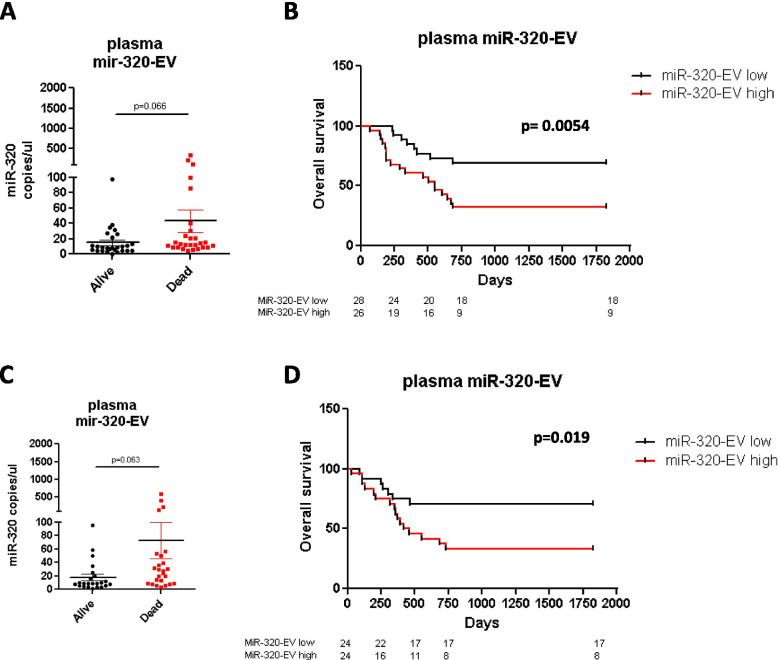
Kaplan–Meier curves reporting the overall survival of lung cancer patients stratified by miR-320 levels inside EVs. **A)** Graphs show miR-320 levels in EVs isolated from lung cancer patients alive (*n* = 27) or dead (*n* = 27) after five years of follow up in the training set. **B)** Analysis of the overall survival of 54 lung cancer patients stratified considering the median of miR-320 copies. **C)** miR-320 copies inside EVs isolated from lung cancer patients alive (*n* = 24) or dead (*n* = 24) after five years of follow up in the validation cohort. **D)** Curves confirmed the prognostic value of circulating miR-320-EVs in lung cancer patients (*n* = 48)

In order to explore the role of both miR-320-EV and miR-126-EV as prognostic biomarkers in lung cancer patients, we initially analyzed their copy number by digital PCR in plasma-EVs collected before surgery in a training set of 54 lung cancer patients (Suppl. Table 3). As showed in Fig. 6A, we noticed enrichment of miR-320 in plasma-EVs from patients with a worse prognosis (average miR-320 copies/µl = 44 ± 14 for dead patients compared to 15.3 ± 3.7 in patients alive at 5 years *p* = 0.066) compared to patients still alive at 5 years, whereas no differences were found in the copy number of miR-126 inside EVs (Suppl. Figure 9A). No correlation was observed between miRNAs levels and tumors stage. In order to investigate the role of these EVs as prognostic biomarkers, we clustered our patients as high and low for EV-miRNAs expression based on the median number of copies of miR-320 or miR-126 inside EVs (10.9 and 78 copies/ul for miR-320-EV and miR-126-EV respectively) in the training cohort. As showed by Kaplan–Meier analysis (Fig. 6B) we noticed that higher levels of plasma miR-320-EV were significantly associated with poor overall survival (*p* = 0.0054) with a hazard ratio (HR) of 2.96. On the contrary, no statistical association between miR-126-EV level and lung cancer patient’s prognosis was observed (Suppl. Figure 9B).

To test the consistency of our findings we tested miR-320-EV and miR-126-EV in a validation set of 48 lung cancer patients with the same clinical characteristics (Suppl. Table 3). We confirmed that miR-320 levels are higher in EVs isolated from lung cancer patients with worse prognosis (Fig. 6C, *p* = 0.063) and observed a significant association between miR-320-EV high and short overall survival [HR 2.68] (Fig. 6D, *p* = 0.019). MiR-126-EV did not show prognostic value also in the validation cohort (Suppl. Figure 4C-D). These results suggest that miR-320-EV could have a direct role in lung cancer progression and is also suitable as a prognostic marker.

## Discussion

Several studies have revealed that cancer progression is driven by a complex systemic interplay between tumor's genetic alterations and paracrine effectors within the host microenvironment, thus, the knowledge of these cellular mechanisms is fundamental for early detection and treatment of lung cancer. Supporting cells in the lung microenvironment, such as lung fibroblasts, endothelial cells and circulating immune cells can control several epithelial cell processes, such as proliferation, metabolism and apoptosis. Interestingly, these processes are finely regulated by the concentration of oxygen in the microenvironment. Indeed, hypoxic conditions enhance EVs production by stromal cells [35], improving local and distant cell-to-cell communication and alter the cargo (miRNAs or proteins) of stromal-derived EVs [35, 36].

In the present study, we focused on the role of circulating plasma EVs and in particular on their miRNA cargo associated with a high-risk to develop lung cancer, to understand their role during lung carcinogenesis.

We previously described that reciprocal ratios of 24 circulating miRNAs predicts lung cancer occurrence up to two years before CT-detection of lung nodules and are able to identify tumor aggressiveness [18]. These signatures were also able to predict response to immunotherapy in advanced lung cancer patients [37]. MiRNAs modulation in plasma reflected in the bloodstream phenotypic changes occurred in several cellular components of the lung microenvironment and represented powerful biomarkers of lung cancer development [19]. Furthermore, we checked the presence of 24 miRNAs inside EVs showing a good correlation with their relative plasma levels in heavy-smokers with different risk of lung cancer [38] suggesting a potential use of EV-miRNAs released by the lung microenvironment as biomarkers of lung cancer risk. EVs and their content could represent interesting biomarkers for cancer detection but all the published studies investigated the importance of cancer-derived EVs as diagnostic tools [39, 40].

Several studies indicate the importance of tumor-derived EV-miRNAs in lung cancer development [41, 42] but no works described the role of stromal-derived EVs from healthy individuals in the promotion of lung tumorigenesis. We demonstrated, for the first time, that different miRNAs cargo in EVs released from healthy individuals with high risk of lung cancer has a protumorigenic role in the promotion of lung cancer. In particular, high levels of miR-126 in MSCpos subjects are able to stimulate the activation of pro-angiogenic mechanisms in endothelial cells. Furthermore, we demonstrated that miR-320 that is up-regulated in plasma MSCpos-EVs and also in EVs from PMN of high-risk individuals induced an immunosuppressive phenotype of macrophages. These data highlight the role of miRNAs in plasma EVs in the modulation of lung microenvironment through a protumorigenic and immunosuppressive phenotype.

Analysis of plasma-derived EVs from heavy smokers unveiled the presence of several surface proteins, such as HLA, CD45, along with specific markers of blood cell subset as CD14, CD11c, CD56 and CD3. Importantly, in our study, CD31 was downregulated in EVs from MSCpos individuals compared to EVs from MSCneg individuals. The loss of CD31 expression on EVs membranes could be considered an early biomarker for hypoxia [43]. Moreover, HLA-DR that has also been described as a specific marker of EVs produced by the Th1 subset of T cells [44] is lower in MSCpos-EVs, potentially reflecting a decrease in EVs released by activated T cells and an increased production by myeloid-derived suppressor cells. These data suggest that circulating EVs may reflect the activation or suppression of specific immune cells during lung carcinogenesis.

In a mouse model of alveolar hypoxia, the release of proangiogenic factors that promote lung tumor growth, such as VEGF-A and an increase of C-Myc in lung tissues was observed [33]. We demonstrated that under hypoxic stimulation, c-Myc levels in fibroblasts were upregulated and released in fibroblast-derived EVs. Importantly, delivery of this transcription factor can stimulate the proliferation of epithelial cells through the modulation of miR-92a and the TGF-β receptor axis accordingly to our previous paper [32]. Hypoxia enhances rapid and chaotic blood vessel formation during tumorigenesis [45]. In high-risk individuals, endothelial cells under hypoxic conditions upregulate miR-126, a key mediator of angiogenesis [29], and its loading into EVs, which induces the production of VEGF through an autocrine loop.

Furthermore, the protumorigenic PMN phenotype has been demonstrated to be induced by TGF-β release under hypoxic conditions [46]. PMNs isolated from MSCpos heavy smokers exhibited increased transport of miR-320 in EVs and induced an M2-like phenotype that was mediated by transfer of miR-320 from PMN-derived EVs into macrophages. MiR-320 present in microvesicles-derived epithelial cells, has already been linked to the modulation of the proinflammatory activity of macrophages [31].

Smoke and hypoxia may act synergistically to generate an immunosuppressive and pro-tumorigenic microenvironment that leads to tumor development. Cigarette smoke can enhance the production of inflammatory factors and reactive oxidative species and induce the activation of HIF and, downstream effectors [47]. VEGF-A is an important mediator of lung inflammation[47] and a mitogen in the endothelium [48]. Therefore, changes in VEGF-A expression according to the chronic obstructive pulmonary disease (COPD) grade could be involved in pulmonary vascular remodeling at early stages of the disease [49].

Our data suggest that the alteration in miRNAs levels observed in EVs from high-risk smokers could reflect changes in lung stromal cells that are able to generate a pro-tumorigenic microenvironment and sustain tumor growth. Chronic cigarette smoke exposure in subjects with a miRNA –based high risk profile induces intracellular alterations in the lung microenvironment and, consequently, in the EVs released by these cells.

We are aware that miRNAs are a minor component of EV-cargo [50], so other components such as proteins, which may play a role in the mediation of EVs pro-tumorigenic effects in concert with miRNAs, will be evaluated in future studies.

EVs and their miRNAs content gained attention as diagnostic tool for lung cancer detection or for prognosis of patients. An interesting study showed that 27 miRNAs were present in lung cancer-derived compared to EVs isolated from healthy volunteers [51]. Interestingly, the authors found the upregulation of miR-320a and miR-126 in tumor derived-EVs without showing any analyses regarding the prognostic role of these miRNAs in lung cancer patients. Here, high levels of miR-320 in plasma-EVs of lung cancer patients were associated with poor prognosis and shorter overall survival.

## Conclusion

In conclusion, based on in vitro experiments, hypoxia-induced modulation of the miRNA cargo of stromal-derived EVs generates a pro-tumorigenic and immunosuppressive microenvironment that stimulates the proliferation and growth of premalignant epithelial cells to promote lung tumor development. In this context, circulating EVs enriched in miR-320 play a key role by inducing immunosuppressive features and could also be an useful prognostic biomarker in lung cancer patients.

## Supplementary Information

**Additional file 1:**

**Additional file 2:**

## Data Availability

Data are available from the corresponding author on reasonable request.

## Abbreviations

COPD: Chronic obstructive pulmonary disease
EV: Extracellular Vesicles
HUVEC: Human Umbilical Vein Endothelial Cells
LDCT: Low-dose computed tomography
MILD: Multicentric Italian Lung Detection
MISEV: Minimal Information for Studies of Extracellular Vesicles
MSC: microRNA signature classifier
NSCLC: Non-small-cell lung cancer
PMN: Polymorphonuclear cells
TEM: Transmission Electron Microscopy
TGF-β: Tumor Growth Factor β
TME: Tumor MicroEnvironment
VEGF-A: Vascular Endothelial Growth Factor-A
