# Supplementary material for: Circulating extracellular vesicles from individuals at high-risk of lung cancer induce pro-tumorigenic conversion of stromal cells through transfer of miR-126 and miR-320
Source: J Exp Clin Cancer Res. 2021 Jul 21;40:237. doi: 10.1186/s13046-021-02040-3 (PMC8293562; doi:10.1186/s13046-021-02040-3)
Supplement: Supplementary file 1 — Additional file 1: [file 13046_2021_2040_MOESM1_ESM.pptx]

## Slide 1
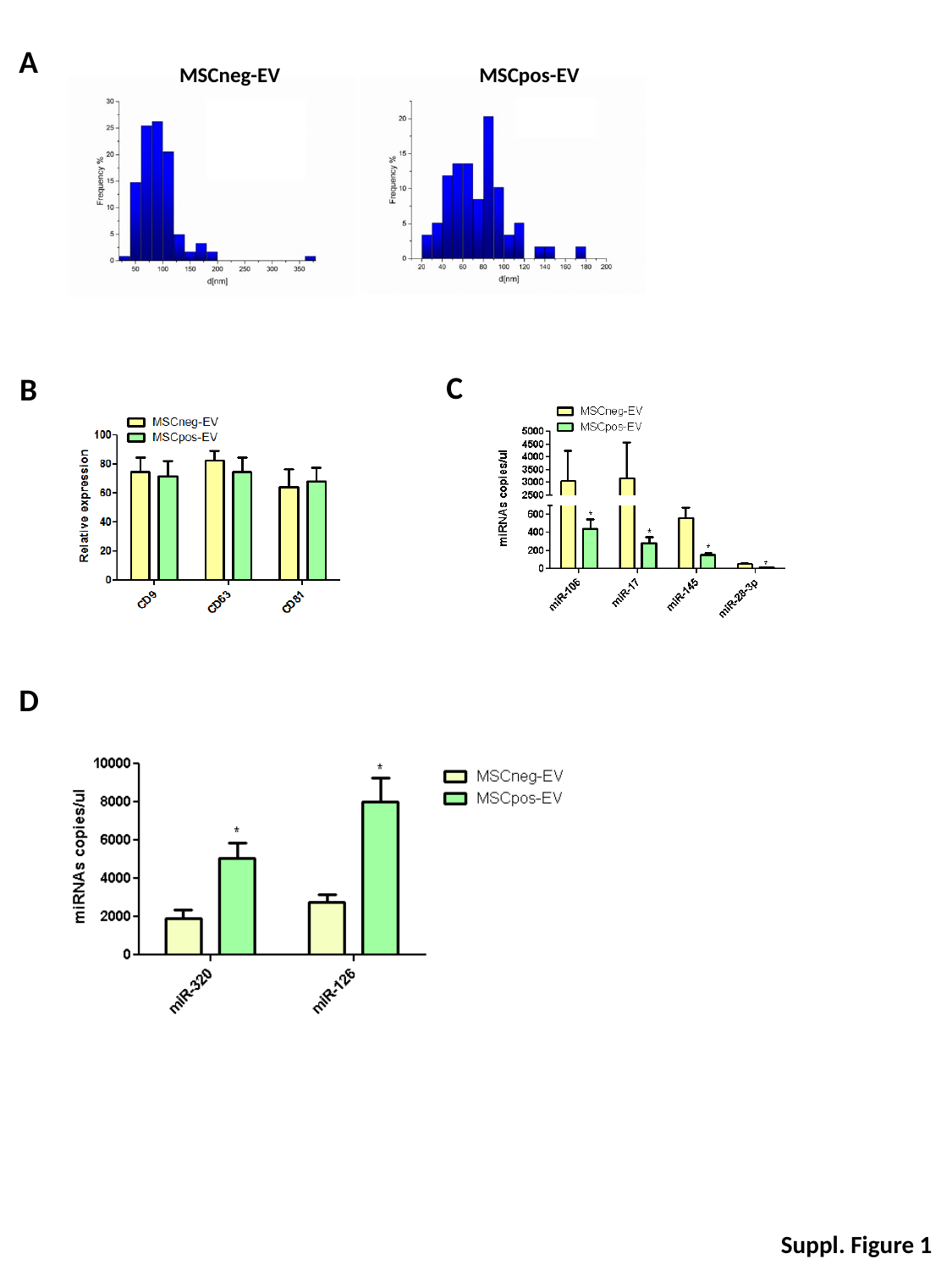

A
MSCneg-EV
MSCpos-EV
C
B
D
Suppl. Figure 1

## Slide 2
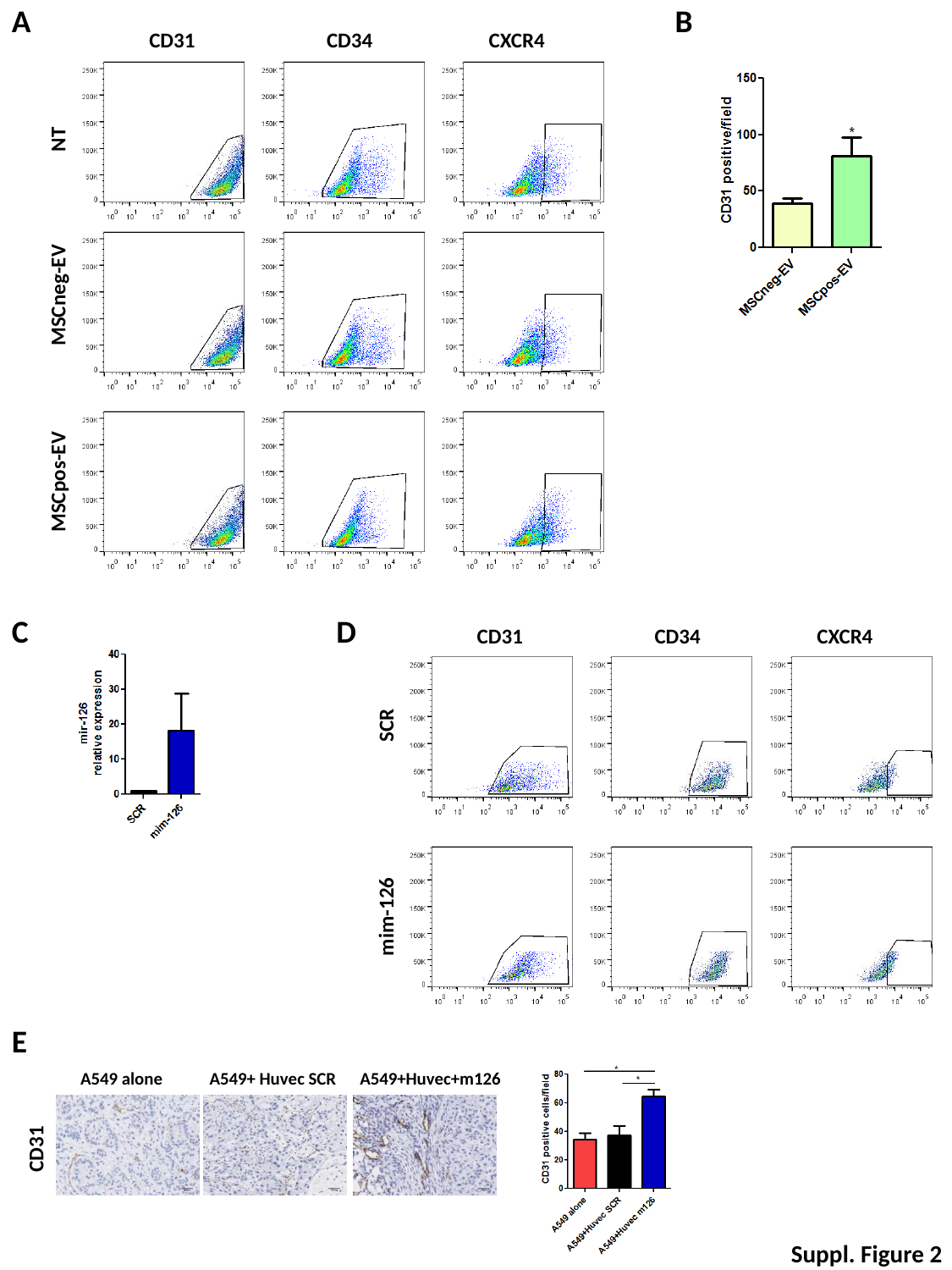

A
B
CD31
CD34
CXCR4
 NT
MSCneg-EV
MSCpos-EV
C
D
CD31
CD34
CXCR4
SCR
mim-126
E
A549 alone
A549+ Huvec SCR
A549+Huvec+m126
CD31
Suppl. Figure 2

## Slide 3
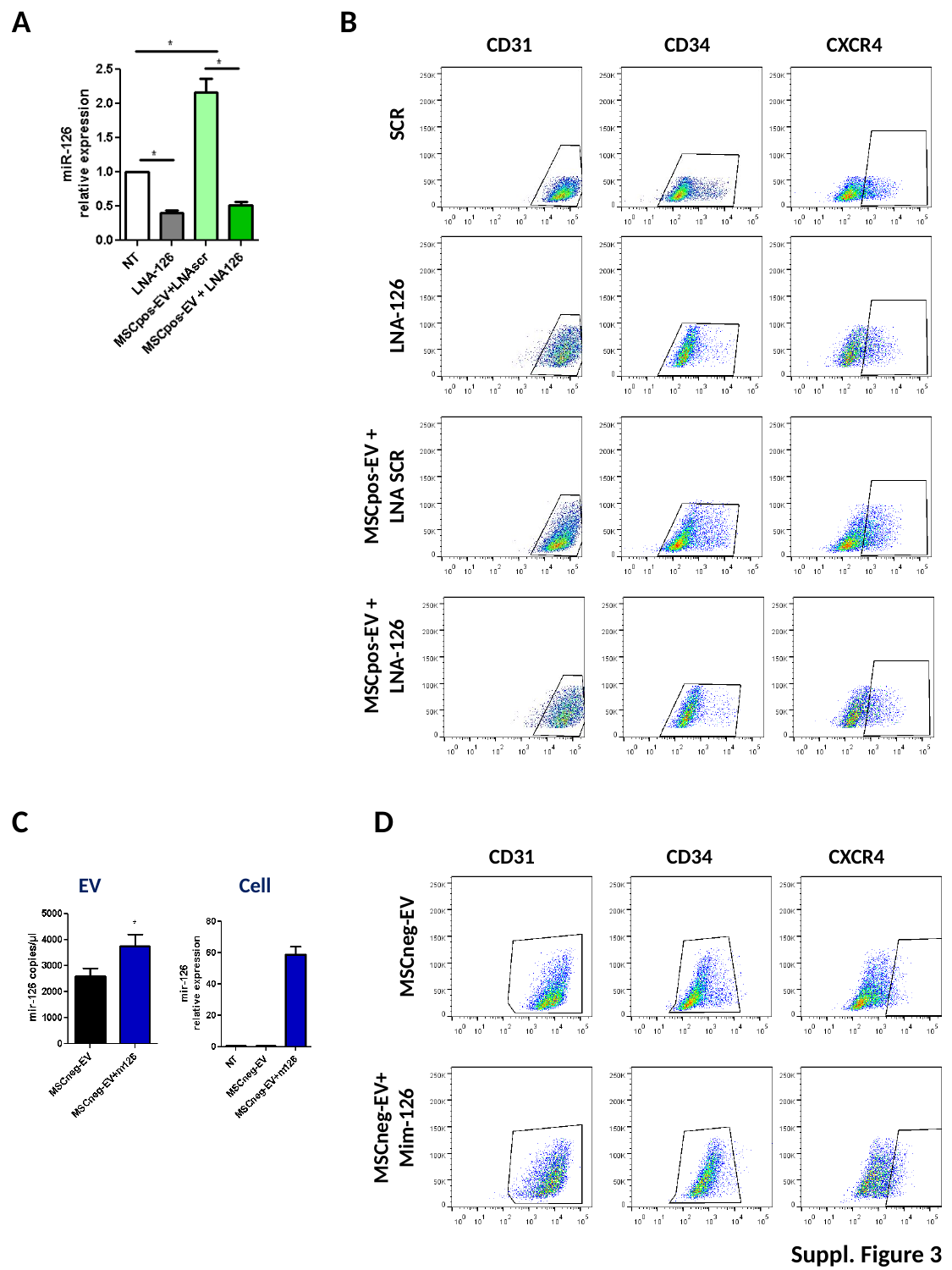

A
B
CD31
CD34
CXCR4
SCR
LNA-126
MSCpos-EV +
LNA SCR
MSCpos-EV +
LNA-126
D
C
CD31
CD34
CXCR4
EV
Cell
MSCneg-EV
MSCneg-EV+
Mim-126
Suppl. Figure 3

## Slide 4
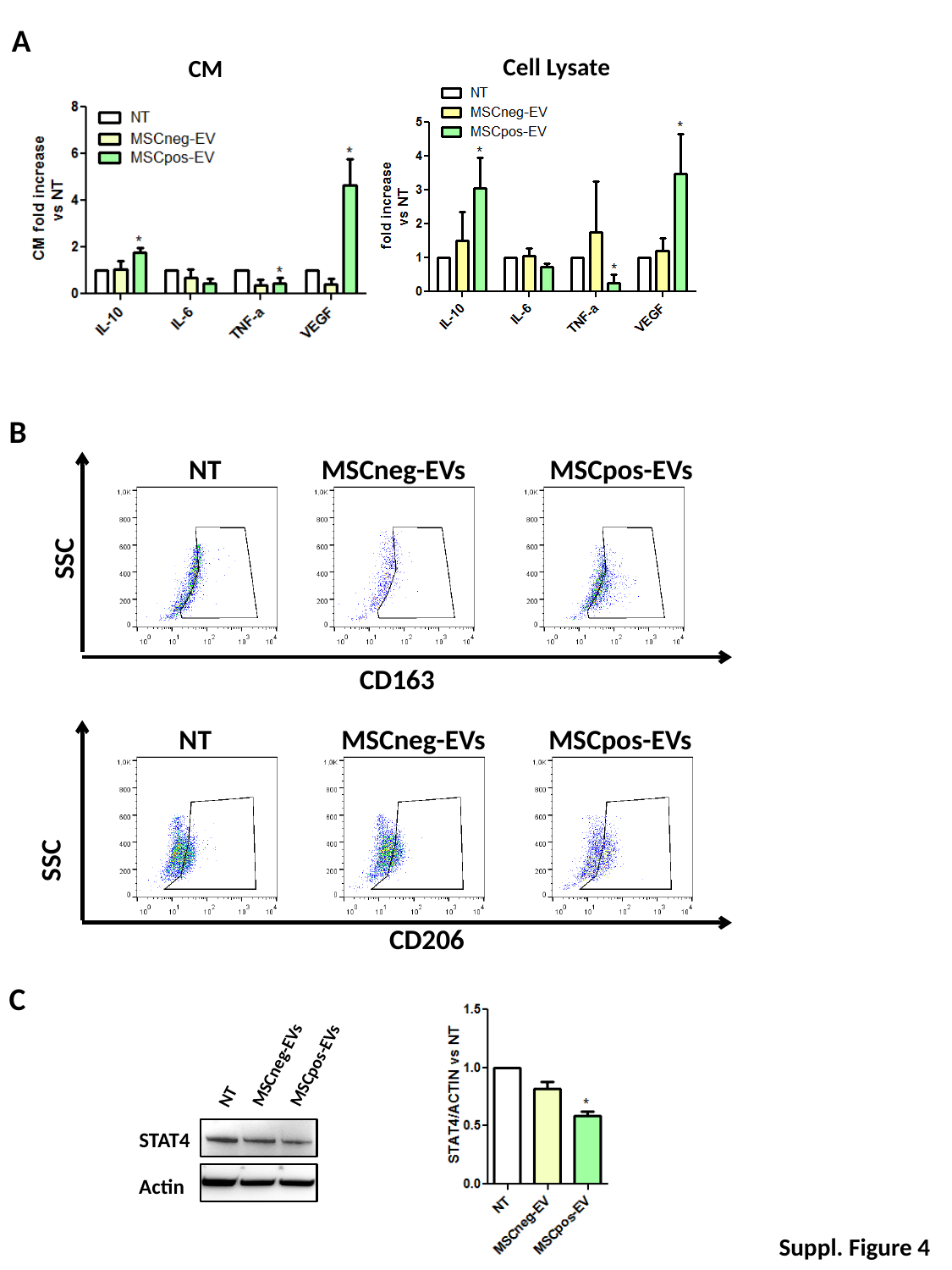

A
Cell Lysate
CM
B
NT
MSCpos-EVs
MSCneg-EVs
SSC
CD163
NT
MSCneg-EVs
MSCpos-EVs
SSC
CD206
C
MSCneg-EVs
MSCpos-EVs
NT
STAT4
Actin
Suppl. Figure 4

## Slide 5
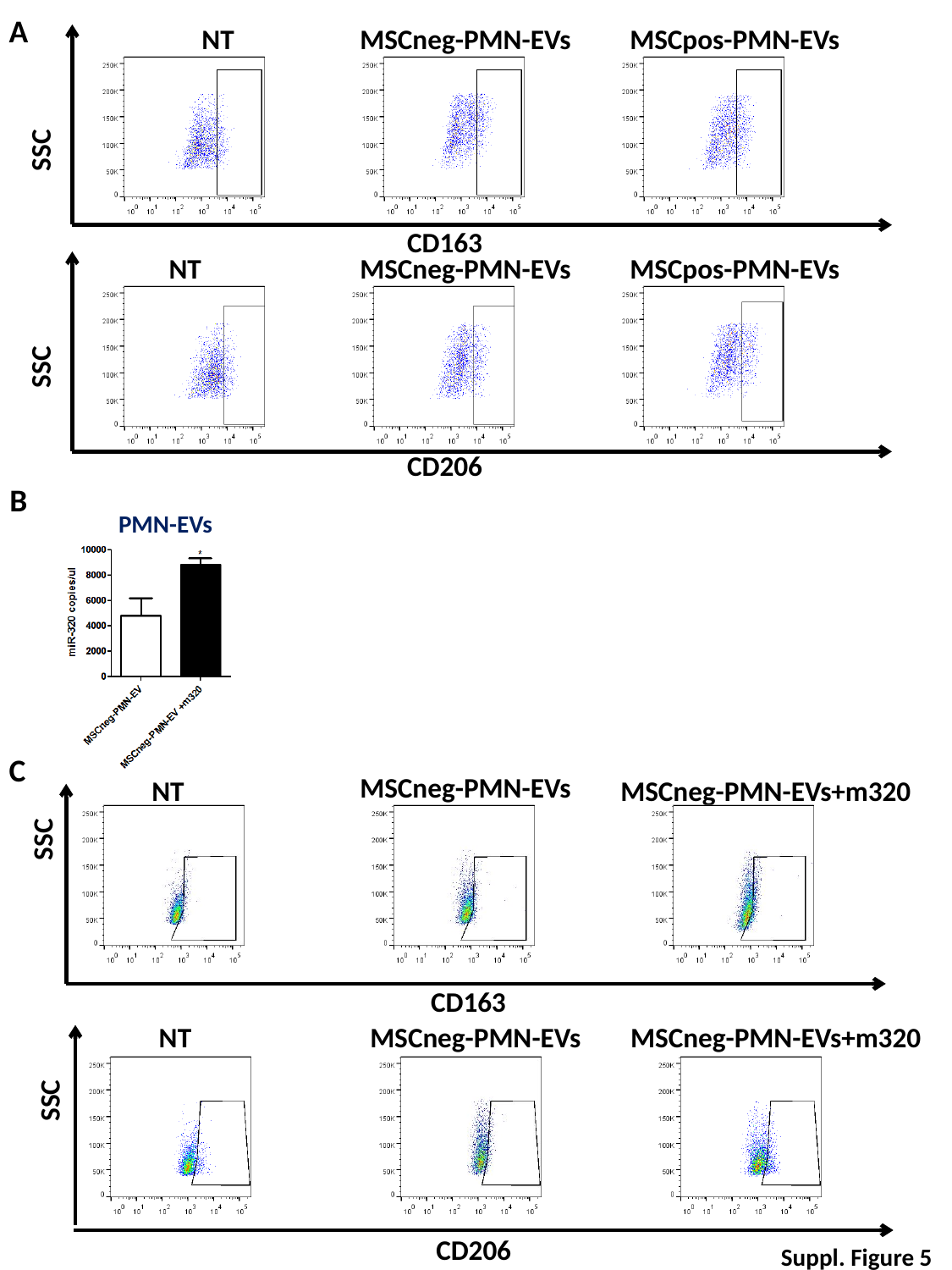

A
NT
MSCpos-PMN-EVs
MSCneg-PMN-EVs
SSC
CD163
NT
MSCneg-PMN-EVs
MSCpos-PMN-EVs
SSC
CD206
B
PMN-EVs
C
MSCneg-PMN-EVs
NT
MSCneg-PMN-EVs+m320
SSC
CD163
NT
MSCneg-PMN-EVs
MSCneg-PMN-EVs+m320
SSC
CD206
Suppl. Figure 5

## Slide 6
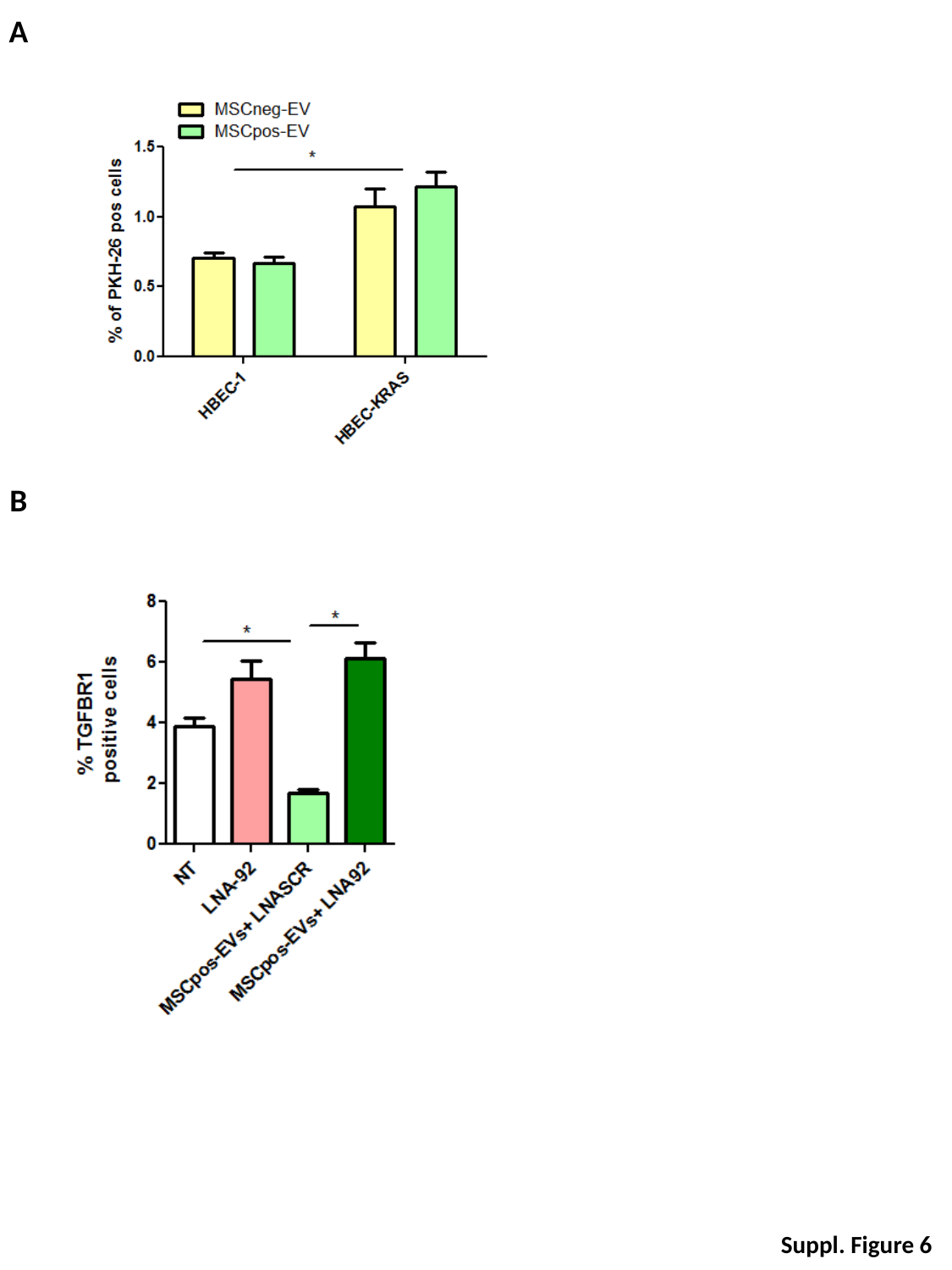

A
B
Suppl. Figure 6

## Slide 7
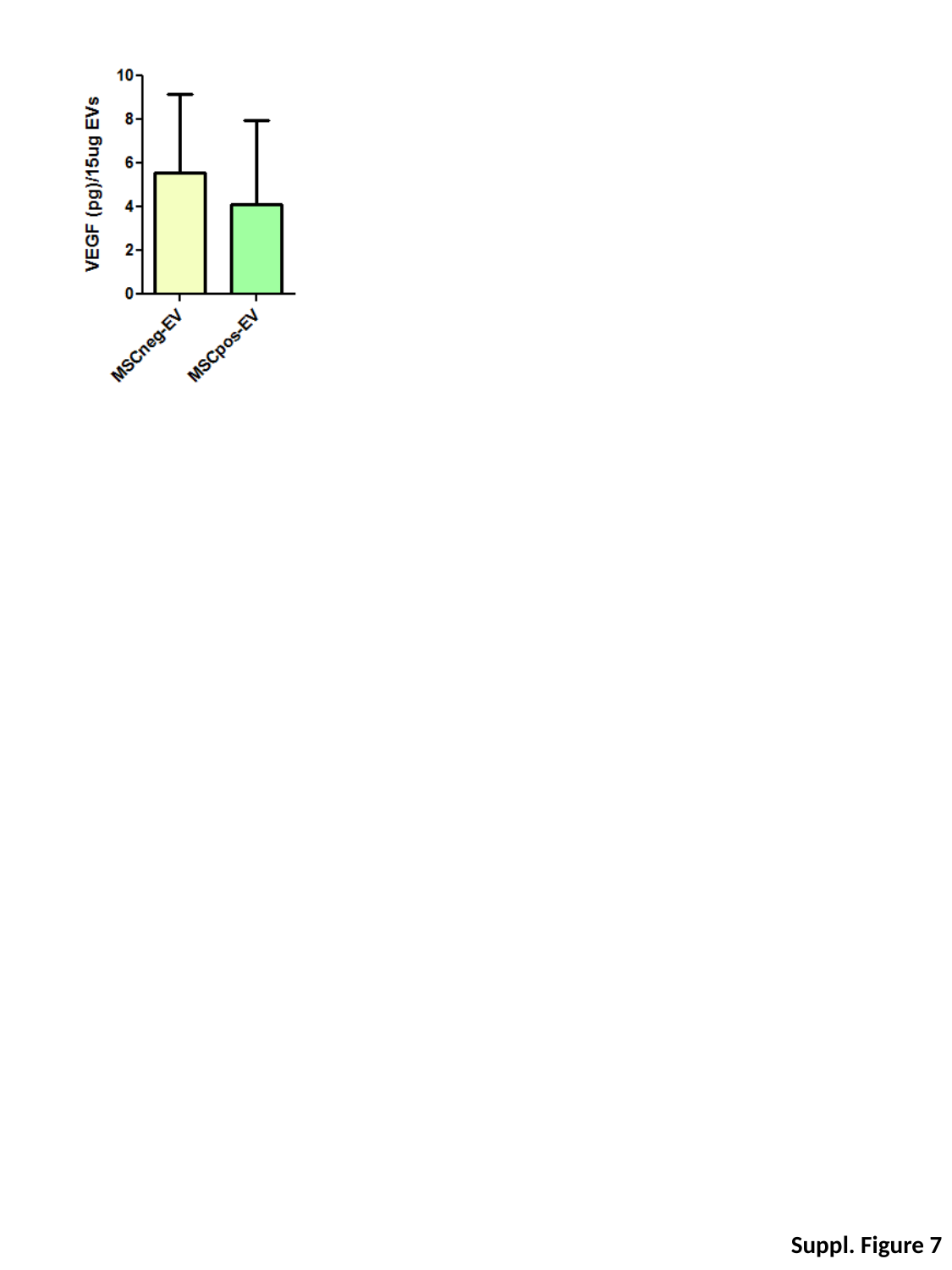

Suppl. Figure 7

## Slide 8
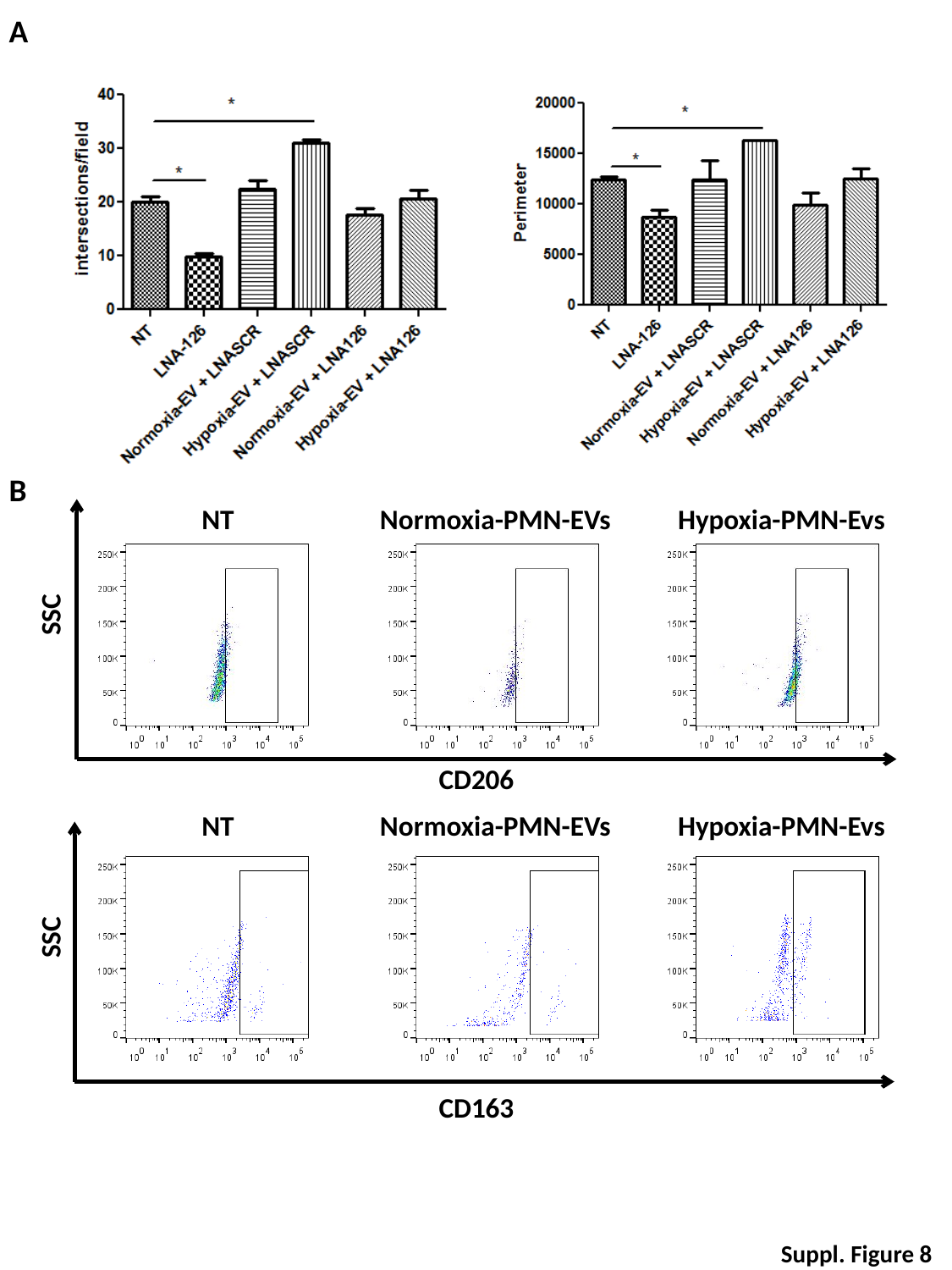

A
B
NT
Hypoxia-PMN-Evs
Normoxia-PMN-EVs
SSC
CD206
NT
Hypoxia-PMN-Evs
Normoxia-PMN-EVs
SSC
CD163
Suppl. Figure 8

## Slide 9
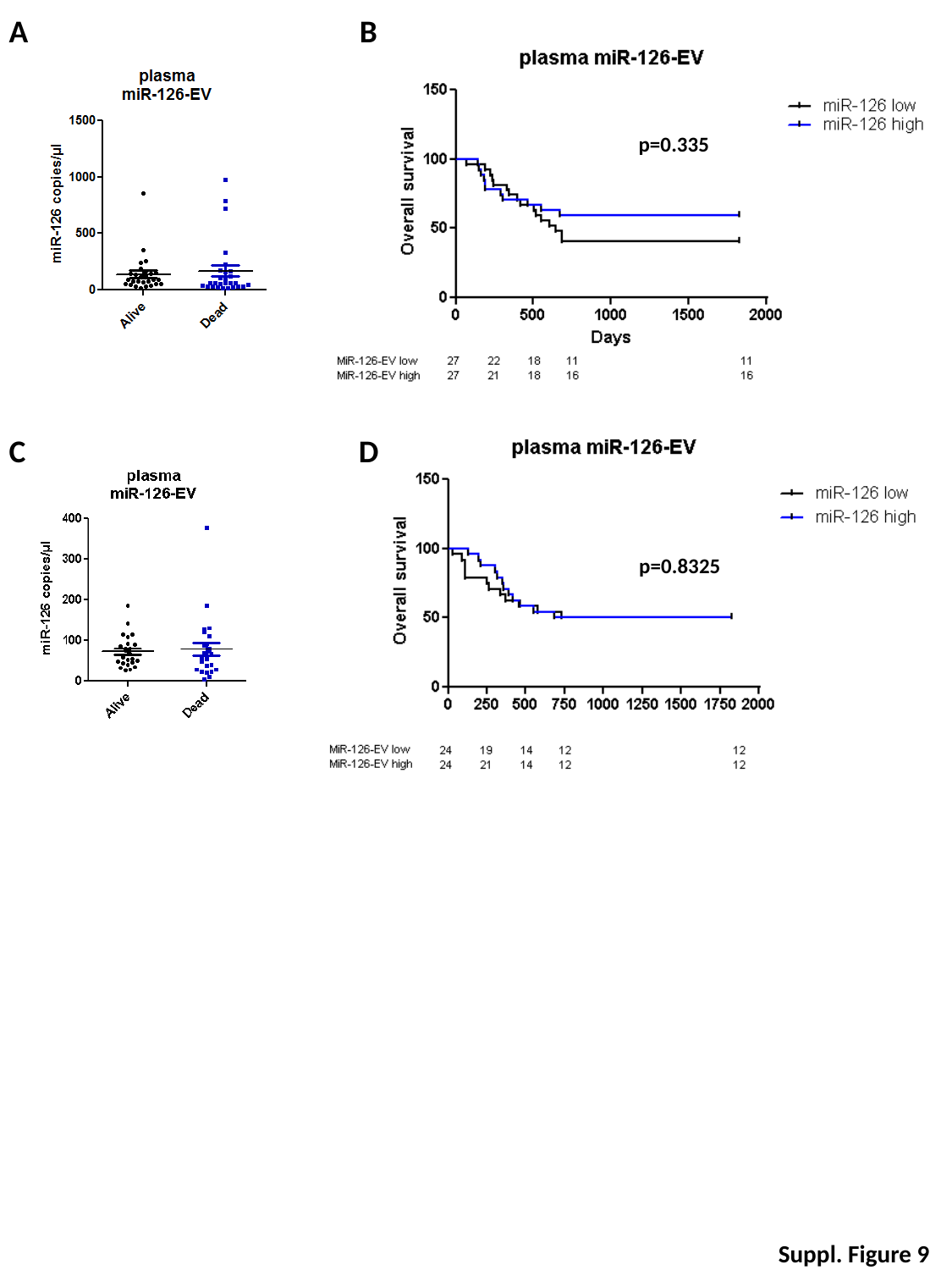

A
B
p=0.335
C
D
p=0.8325
Suppl. Figure 9
