# Supplementary material for: Circulating extracellular vesicles from individuals at high-risk of lung cancer induce pro-tumorigenic conversion of stromal cells through transfer of miR-126 and miR-320
Source: J Exp Clin Cancer Res. 2021 Jul 21;40:237. doi: 10.1186/s13046-021-02040-3 (PMC8293562; doi:10.1186/s13046-021-02040-3)
Supplement: Supplementary file 2 — Additional file 2: [file 13046_2021_2040_MOESM2_ESM.docx]

Material and Methods

*Cell culture*

Immortalized bronchial epithelial HBEC-1 and HBEC-KRAS^V12high^ cells were provided by Prof. J.D. Minna (UT Southwestern, TX) as previously described [1]. These cells were cultured in Keratinocyte–Serum-Free Medium (K-SFM; Thermo Fisher Scientific, Waltham Massachussetts, USA) supplemented with 5 ng/ml human recombinant EGF and 50 μg/ml bovine pituitary extract (Thermo Fisher Scientific). The lung cancer cell line A549 was purchased by American Type Colture Collection and cultured in RPMI 1640 medium (Gibco, Thermo Fisher Scientific, Waltham, MA, USA) supplemented with 10% fetal bovine serum (FBS; EuroClone, Italy) and 2% penicillin-streptomycin (Sigma-Aldrich, Saint Louis, MO, USA). Primary human umbilical vein endothelial cells (HUVECs) were purchased from Lonza (Lonza, Basel, Switzerland) and cultured in Endothelial Cell Growth Basal Medium-2 (EBM-2; Lonza, Basel, Switzerland). Primary cancer-associated fibroblast lung cancer (CAF-154-hTERT) cells were isolated in our laboratory and characterized for the expression of mesenchymal markers (CD90, CD105, CD73, and CD166) and activation markers (alpha-SMA and FAP) to confirm their stromal origin, as previously described [2]. Monocytes were isolated from PBMC samples by plastic adherence and differentiated into macrophages by culture for 7 days in RPMI supplemented with 10% FBS and 50 ng/ml M-CSF (Sigma-Aldrich, Saint Louis, Missouri, USA). Cell lines were cultured in a humidified incubator containing 5% CO_2_ at 37°C. All immortalized cell lines were authenticated by DNA short tandem repeat (STR) profiling and confirmed to be mycoplasma-negative.

For all *in vitro* hypoxia experiments, cells were maintained in an incubator containing 2% oxygen at 37°C for 24 h.

*EVs isolation*

Plasma EVs were obtained from whole blood as previously described [3]. Plasma and conditioned medium (CM)-derived EVs were purified by differential centrifugation processes as previously described [4](. In brief, EVs were isolated from 1 ml of stored plasma by ultracentrifugation at 120000 × g and 4°C for 90 minutes using a TLA-100.3 fixed-angle rotor in a TL-100 ultracentrifuge (Beckman Coulter, Brea, CA, USA). To remove impurities, the EV-enriched pellet was washed in phosphate-buffered saline (PBS; Thermo Fisher Scientific) at 120000 × g for 60 minutes at 4°C. Then, the pellet was resuspended in PBS or directly lysed in RIPA buffer (Sigma-Aldrich) supplemented with protease and phosphatase inhibitors and was stored at −80°C. The protein content of the purified EVs was determined by the Bradford assay.

For isolation of CM-derived EVs, the standard medium was replaced with serum-free medium 48 h before the cells were 70-80% confluent. EVs were isolated from 70 ml of CM for each condition. CM was centrifuged first at 300 × g for 10 minutes and then at 3200 × g for 25 minutes to remove cell debris. Then, the supernatant was filtered through 0.22-μm filters (Millipore, Burlington, MA, USA) to exclude large vesicles prior to ultracentrifugation according to the procedure described above for plasma-derived EVs.

For isolation of hypoxia-derived EV, cells were maintained in an incubator containing 2% oxygen at 37°C, and EVs were isolated from CM as described above after 24 h.

The EV concentration and size distribution were determined by using a NanoSight NS300 instrument (Malvern Panalytical) as described in [4]. The videos were analyzed using NTA 3.2 software.

*Treatment with EVs*

For all *in vitro* treatments with plasma- or CM-derived EVs, 5×10^4^ cells/well (HBEC-KRAS^V12high^, CAFs, HUVECs, PMNs or macrophages) were seeded in triplicate wells in 6-well plates and treated with 15 μg of EVs.

To assess EV uptake *in vitro*, we used plasma-derived EVs labeled with PKH26, as we previously described [4]. We seeded cells (5 × 10^4^ cells/well) in a 6-well plate and treated them with 1 μg of PKH26-labeled EVs. After 24 h, cells were harvested, centrifuged, resuspended in PBS and analyzed in a FACSCanto II (BD Biosciences, San Jose, CA, USA).

*Transmission Electron Microscopy*

EV morphology was assessed as previously described using a Zeiss LIBRA 200FE transmission electron microscope with an in-column second-generation Omega filter [4]. The size of the EVs was estimated by measuring one hundred EVs using the iTEM transmission electron microscopy (TEM) imaging platform (Olympus).

*Western blotting*

Western blotting was performed with an SDS–PAGE system. Proteins were extracted from cells and EVs with RIPA buffer. Then, 40 μg of protein extract was loaded on a Bolt 4–12% Bis- Tris gel (Thermo Fisher Scientific) and transferred to a PVDF membrane, which was then incubated with the following antibodies: anti-CD9 (Cell Signaling; 1:1000), anti-CD81 (Thermo Fisher Scientific; 1:100), anti-Alix (BioLegend; 1:1000), anti-STAT4 (C46B10, 1:1000, Cell Signalling), anti-c-Myc (Cell Signaling, 1:1000) and mouse anti-actin (1:2000, Sigma Aldrich) primary antibodies and the corresponding anti-mouse and anti-rabbit peroxidase-conjugated secondary antibodies (GE Healthcare Life Sciences, 1:2000). Signals were detected via an enhanced chemiluminescence (ECL, GE Healthcare) reaction in a MINI HD9 Western Blot Imaging System (Cleaver Scientific Ltd, United Kingdom). Western blot band densities were quantified using ImageJ software.

*Flow cytometry*

Flow cytometric analysis was performed with 30 μg of EVs in accordance with the procedures previously described by Théry et al. [5].

For characterization of plasma-derived EVs, EVs were first incubated with 10 μl of latex beads for 15 minutes at room temperature (RT) and then incubated overnight at 4°C in PBS. After incubation, 100 mM glycine was added to each sample for 30 minutes at RT, and the samples were then stained with 1 μg of the following primary antibodies (Ab): anti-CD9, anti-CD81 (Cell Signaling, Danvers, Massachusetts, USA), anti-CD63 (Abcam, Cambridge, UK), and anti-c-Myc (Cell Signaling) for 30 minutes at 4°C. Then, the samples were incubated for 30 minutes at 4°C with their corresponding fluorescent secondary antibodies: Alexa Fluor 647-conjugated goat anti-rabbit IgG (Thermo Fisher Scientific) or DyLight 488-conjugated goat anti-mouse IgG (Bethyl). Samples were analyzed via flow cytometry (FACSCanto II, BD Biosciences, San Jose, CA, USA) and FlowJo software (TreeStar, Ashland, OR, USA).

The following antibodies were used: macrophage-specific CD163-PE and CD206-FITC (Biolegend, San Diego) and HUVEC-specific CD31-FITC, CXCR4-APC and CD34-PE (Biolegend, San Diego). Cells were stained for 15 minutes at room temperature in the dark and analyzed using a FACSCanto II (BD Biosciences, San Jose, CA, USA) with FlowJo software.

*MACSPlex multiplex EV analysis*

Surface marker profiling of EVs isolated from CM from fibroblasts, endothelial cells, polymorphonuclear leukocytes (PMNs) and epithelial cells was performed using a MACSPlex Exosome Kit in accordance with the manufacturer’s instructions (Miltenyi Biotec, Bergisch-Gladbach, Germany). In brief, 15 μl of MACSPlex Exosome Capture Beads (previously vortexed) was added to each sample (15 μg), and 15 μl of MACSPlex Exosome Detection Reagent cocktail (CD9, CD63, and CD81) was added to each tube and mixed by pipetting. Samples were incubated at 4°C in the dark for 1 h, washed two times with, 500 μl of MACSPlex Buffer, and analyzed by flow cytometry (FACSCanto II, BD Biosciences, San Jose, CA, USA) and FlowJo software (TreeStar, Ashland, OR, USA).

*MiRNA expression analysis*

Total RNA was extracted from EVs and cells using a Maxwell RSC Instrument (Promega, Madison, WI, USA) and a Maxwell RSC miRNA Tissue Kit (Promega) in accordance with the manufacturer’s instructions. Then, miRNAs were reverse transcribed using a TaqMan microRNA Reverse Transcription Kit and a TaqMan RT Primer Pool specific for the miRNAs of interest according to the manufacturer’s instructions (Thermo Fisher Scientific), as previously described [3]. MiRNA expression was then evaluated using the TaqMan assay (Thermo Fisher Scientific) and normalized to the expression of the small nucleolar RNA RNU48.

For absolute quantification of the miRNA content in EVs, 15 μg of EVs was treated with RNase A (10 μg/ml; Roche, Basel, Switzerland) for 30 minutes before RNA extraction to degrade miRNAs not contained in EVs. Total RNA was extracted and analyzed as described above, and synthetic *Caenorhabditis elegans* miRNA-39 (cel-miR-39) was used as the spike-in control (25 fmol) in each sample [6]. The miRNA content was evaluated using chip-based digital PCR (dPCR) (Thermo Fisher Scientific) as described by Conte et al. [7].

*Matrigel assay*

In brief, 2×10^4^ EV-pretreated HUVECs were added to 8-well chamber slides precoated with 150 µl of Matrigel (Becton Dickinson) in a total volume of 150 µl of EGM-2. After 5 h of incubation at 37°C, capillary-like structures were fixed with 2% paraformaldehyde. The assays were performed in duplicate wells. Tube formation was quantified by counting the number of intersection points (20× magnification) and network perimeters (4× magnification) in the 5 random fields of view imaged.

*miRNA transfection*

HUVECs (5 × 10^4^ cells/well) were seeded in a 6-well plate and transfected with miR-126 mimics (mim-126) or negative control (SCR) (50 nM, Thermo Fisher Scientific) with Lipofectamine 2000 (Thermo Fisher Scientific) according to the manufacturer’s protocols. Transfection of the locked nucleic acid (LNA) inhibitor (50 nM; LNA-126, Exiqon, Vedbæk, Denmark) was performed 24 h before EV treatment.

Macrophages were transfected with mirVana miRNA miR-320 mimics (50 nM) as described above. To assess the role of miR-320 in these cells miRCURY LNA inhibitor (50 nM, LNA-320, Exiqon Denmark) or scrambled LNA was transfected into macrophages 24 h before the addition of CM from PMNs.

To assess the role of c-Myc transfer and TGFBRI down-modulation in HBEC-KRAS^V12high^, transfection of LNA inhibitor for mir-92a (50 nM, LNA-92, Exiqon Denmark) was performed 24h after EV treatment.

*Real-Time PCR (RT-PCR)*

cDNA was synthesized from 250 ng of extracted total RNA. RT‐PCR was performed using TaqMan Universal Master Mix II (Thermo Fisher Scientific) according to the manufacturer’s instructions. Relative quantification of the expression levels of the selected genes was performed using GAPDH or B2M as the endogenous control.

*Proliferation assays*

HBEC-KRAS^V12high^ cells were treated with 15 μg of EVs for 24 h. Pretreated HBEC-KRAS^V12high^ cells (5 × 10^3^ cells/well) were plated in 96-well plates, and a RealTime-Glo assay (Promega) was performed after 72 h in accordance with the manufacturer’s instructions. Moreover, proliferation under the same conditions was assessed by cell counting with a Trypan blue exclusion dye assay after 72 h of treatment.

The 3D proliferation assay was performed using VitroGel 3D (TheWell Bioscience, USA) in accordance with the manufacturer’s instructions. A 1:1 VitroGel mix was added to 24-well plates and incubated for 20 minutes at 37°C. Then, pretreated HBEC-KRAS^V12high^ cells (5 × 10^4^ cells/well) were plated on the VitroGel. After 14 days, colonies were counted in 5 random fields per condition, as described by Borzi et al. [4].

*ELISA*

Total c‐Myc protein in cellular and EV-derived lysates was quantified using a c-Myc human ELISA kit (Thermo Fisher Scientific) in accordance with the manufacturer’s instructions. The absorbance at 450 nm was measured using a microplate reader (Infinite M1000, Tecan).

VEGF secretion in the CM of macrophages and HUVECs was analyzed using a Human VEGF-A ELISA kit according to the manufacturer’s instructions (RayBiotech).

***IHC analysis***

Murine CD31 (PECAM-1) protein was investigated by IHC staining. Briefly sections 2.5/3 micron-thick were cut from paraffin blocks, dried, de-waxed, rehydrated, and unmasked (with Dako PT-link, EnVision™ FLEX Target Retrieval Solution, High ph -15 min - 96°C).  CD31 antibody (clone D8V9E, Cell Signaling Technology, dilution 1:100) was incubated with a commercially available detection kit (EnVision™ FLEX+, Dako, Agilent) in an automated Immunostainer (Dako Autostainer Link 48 - Agilent).

**Suppl. Table 1. Clinical characteristics of heavy-smokers**

|  | MSCneg (n=20) | MSCpos (n=20) |
| --- | --- | --- |
| Gender |  |  |
| Male | 14 (70%) | 14 (70%) |
| Female | 6 (30%) | 6 (30%) |
| Age (years) | 60+6,9 | 64,7+7,7 |
| Smoking status |  |  |
| Current | 16 (80%) | 18 (90%) |
| Former | 4 (20%) | 2 (10%) |
| Smoking habit (Pack-Year index) | 38,7+7,7 | 43,2+8 |

**Suppl. Table 2. Clinical characteristics of lung cancer patients**

|  |  | Training set (n=54) | Validation set (n=48) |
| --- | --- | --- | --- |
| Gender |  |  |  |
|  | **Male** | 38 | 37 |
|  | **Female** | 16 | 11 |
| Age |  | 66+12.6 | 66+10.6 |
| Tumor Histology |  |  |  |
|  | **ADC** | 28 | 24 |
|  | **SCC** | 12 | 11 |
|  | **Other** | 14 | 13 |
| Stage |  |  |  |
|  | **I-II** | 35 | 32 |
|  | **III-IV** | 19 | 16 |
| Prognosis |  |  |  |
|  | **Alive (I-II)** | 18 | 16 |
|  | **Alive (III-IV)** | 9 | 8 |
|  | **Dead (I-II)** | 17 | 16 |
|  | **Dead (III-IV)** | 10 | 8 |

**Suppl. Table 3. Size distribution and quantification of EV by Nanoparticles Tracking Analysis**

|  | **MSCneg- EV** | **MSCpos- EV** | **p- value** |
| --- | --- | --- | --- |
| **Particles mode (nm)** | 127.6+24.9 | 131.6+29.8 | 0.8122 |
| **Particles mean (nm)** | 171.9+24.,2 | 188.8+29.8 | 0.3561 |
| **Particles (x10^9^)/ml** | 11.41±2.2 | 8.87±2.6 | 0.470 |

**SUPPLEMENTARY FIGURE LEGENDS**

**Supplementary Figure 1. Characterization of EVs isolated from the plasma of heavy-smokers individuals. A)** Size distribution analysis by TEM. **B)** Quantification of flow cytometry analysis for CD63, CD81, CD9 in MSCpos and neg-EVs. **C)** Quantification by dPCR of the absolute content of miRNAs in MSCpos and MSCneg-EVs. **D)** miR-320 and miR-126 copies in MSCpos-EVs (n=15) and MSCneg-EVs (n=15)

**Supplementary Figure 2. miR-126-EVs induces pro-angiogenic modulation of HUVEC A)**Representative dot plots showed the gating strategy for HUVEC analysis after EVs treatments. **B)** Quantification of CD31 positive cells in MSC-pos and MSCneg-EVs in mice (n=5). **C)** miR-126 expression levels in HUVEC cells after miRNA mimic-126 transfection (n=3). **D**) Dot plots representative of HUVEC-SCR or miR-126 over-expressing cells. **E**) IHC images and quantification of CD31 positive vessels in subcutaneously tumors (n=5). *p<0.05 versus controls. The data are expressed as the mean ± S.E.M. values.

**Supplementary Figure 3. miR-126 is critical for pro-angiogenic phenotype in endothelial cells. A)** miR-126 relative expression in HUVEC cells pre-treated with LNA-126 upon MSCpos- and neg-EVs treatment (n=4.) **B**) Representative flow cytometry plots for endothelial cell activation **C)** Quantification of miR-126 levels in MSCneg-EVs (**left**) and HUVEC cells (**right**) after treatment with MSCneg-EVs enriched with mimic-126 (n=4). Untreated cells were used as control. **D**) Gating strategy of endothelial cells modulation after MSCneg-mim-126 treatment.

**Supplementary Figure 4. MSCpos-EVs induced M2 phenotype in macrophages. A)** Quantification of M2 markers in Conditioned Medium **(left)** or cell lysates **(right)** of macrophages treated with MSCpos or MSCneg EVs (n=3). Untreated cells were used as control. **B**) Gating strategy for the analysis of CD163 and CD206 by Flow Cytometry on macrophages. **C)** Representative images of WB bands and STAT4 quantification after plasma-EVs treatment (n=3) *p<0.05 versus controls. The data are expressed as the mean ± S.E.M. values.

**Supplementary Figure 5. miR-320-EVs induce M2 switch. A)** Dot plots for the analysis of CD163 and CD206 in macrophages**.** **B**) Absolute quantification of miR-320 levels in MSCneg-PMN-EVs and after miR-320 trasfection inside EVs (n=5). *p<0.05 versus controls. The data are expressed as the mean ± S.E.M. values. **C**) Plots showed the induction of CD163 and CD206 on macrophages’ surface after EVs treatment.

**Supplementary Figure 6. MSCpos-EVs increase HBEC-KRAS^V12high^ proliferation. A)** Flow cytometric analysis of the percentage of PKH26^+^ cells after treatment with PKH26-labeled MSCpos- and MSCneg-EVs (1 µg) (n=3 per group). *p<0.05 versus HBEC-1 cells. B) TGFBRI levels in LNA-92 treated cells after the addition of MSCpos-EVs (n=3) Untreated cells were used as control. * p<0.05 versus NT. Data are expressed as the mean ± S.E.M. values.

**Supplementary Figure 7. VEGF quantification in EVs.** Amount of VEGF in MSCpos or MSC-neg-EVs quantified by ELISA (n=6 for each group). Data are expressed as the mean ± S.E.M. values.

**Supplementary Figure 8. miR-126 is critical for the modulation of HUVEC cells by hypoxia-EVs. A)** Cells pretransfected with LNA 126 were incubated with Hypoxia- or Normoxia-EVs, and capillary-like structure formation was analyzed (n=3). **B**) Gating strategy for the analysis of CD163 and CD206 on macrophages. * p<0.05 versus NT. Data are expressed as the mean ± S.E.M. values.

**Supplementary Figure 9. Kaplan-Meier curves reporting the overall survival of lung cancer patients stratified by miR-126 levels inside EVs. A)** Graphs show miR-126 levels in EVs isolated from lung cancer patients alive (n=27) or dead (n=27) in the training set. **B)** Analysis of the overall survival of 54 lung cancer patients stratified considering the median of miR-126 copies. **C)** miR-126 copies inside EVs isolated from lung cancer patients alive (n=24) or dead (n=24) in the validation cohort. **D)** Curves analyzed the prognostic value of circulating miR-126-EVs in lung cancer patients (n=48).

Reference List

1. Sato M, Larsen JE, Lee W, Sun H, Shames DS et al. (2013) Human lung epithelial cells progressed to malignancy through specific oncogenic manipulations. Mol Cancer Res 11: 638-650.

2. Fortunato O, Borzi C, Milione M, Centonze G, Conte D et al. (2018) Circulating mir-320a promotes immunosuppressive macrophages M2 phenotype associated with lung cancer risk. Int J Cancer

3. Fortunato O, Boeri M, Verri C, Conte D, Mensah M et al. (2014) Assessment of circulating microRNAs in plasma of lung cancer patients. Molecules 19: 3038-3054.

4. Borzi C, Calzolari L, Ferretti AM, Caleca L, Pastorino U et al. (2019) c-Myc shuttled by tumour-derived extracellular vesicles promotes lung bronchial cell proliferation through miR-19b and miR-92a. Cell Death Dis 10: 759.

5. Thery C, Amigorena S, Raposo G, Clayton A (2006) Isolation and characterization of exosomes from cell culture supernatants and biological fluids. Curr Protoc Cell Biol Chapter 3: Unit.

6. Kroh EM, Parkin RK, Mitchell PS, Tewari M (2010) Analysis of circulating microRNA biomarkers in plasma and serum using quantitative reverse transcription-PCR (qRT-PCR). Methods 50: 298-301.

7. Conte D, Verri C, Borzi C, Suatoni P, Pastorino U et al. (2015) Novel method to detect microRNAs using chip-based QuantStudio 3D digital PCR. BMC Genomics 16: 849.
